# Natural products in attenuating renal inflammation *via* inhibiting the NLRP3 inflammasome in diabetic kidney disease

**DOI:** 10.3389/fimmu.2023.1196016

**Published:** 2023-05-05

**Authors:** Yan Wang, Zhun Sui, Mi Wang, Peng Liu

**Affiliations:** ^1^ Department of Nephrology, Peking University People’s Hospital, Beijing, China; ^2^ Shunyi Hospital, Beijing Traditional Chinese Medicine Hospital, Beijing, China

**Keywords:** natural products (NP), diabetic kidney disease (DKD), inflammation, the NLRP3 inflammasome, NF- κB, TXNIP (thioredoxin interacting protein)

## Abstract

Diabetic kidney disease (DKD) is a prevalent and severe complications of diabetes and serves as the primary cause of end-stage kidney disease (ESKD) globally. Increasing evidence indicates that renal inflammation is critical in the pathogenesis of DKD. The nucleotide - binding oligomerization domain (NOD) - like receptor family pyrin domain containing 3 (NLRP3) inflammasome is the most extensively researched inflammasome complex and is considered a crucial regulator in the pathogenesis of DKD. The activation of NLRP3 inflammasome is regulated by various signaling pathways, including NF- κB, thioredoxin—interacting protein (TXNIP), and non-coding RNAs (ncRNA), among others. Natural products are chemicals extracted from living organisms in nature, and they typically possess pharmacological and biological activities. They are invaluable sources for drug design and development. Research has demonstrated that many natural products can alleviate DKD by targeting the NLRP3 inflammasome. In this review, we highlight the role of the NLRP3 inflammasome in DKD, and the pathways by which natural products fight against DKD *via* inhibiting the NLRP3 inflammasome activation, so as to provide novel insights for the treatment of DKD.

## 1 Introduction

Diabetic kidney disease (DKD) is one of the most prevalent and severe microvascular complications of diabetes, and is also the main cause of end stage kidney disease (ESKD) globally. Approximately 30% to 50% of ESKD cases worldwide are attributed to DKD (1). The pathogenesis of DKD is multifaceted, involving metabolic abnormalities, renal hemodynamics changes, oxidative stress, and inflammation, among others.

Initially, metabolic and hemodynamic changes were believed to be the main factors in the development of DKD. However, it is gradually realized that inflammation plays an important role in the development and progression of DKD (2, 3). Chronic exposure to advanced glycation end products (AGEs) stimulates the release of chemokines and cytokines, which can be recognized by Toll-like receptors (TLRs) and nucleotide-binding oligomerization domain-like receptors (NLRs), leading to heightened inflammatory responses. Persistent inflammation ultimately results in the onset of DKD (4).

Natural products include a diverse group of substances extracted from various natural sources such as plants, bacteria, fungi, insects, and even animals, they are valuable sources for drug design and development (5). Accumulative evidence demonstrates that many natural products could suppress systemic and renal inflammation by targeting nuclear transcription factor-κB (NF-κB), NLR family pyrin domain containing 3 (NLRP3) inflammasome, transforming growth factor-β (TGF-β) signaling pathway, and exhibit reno-protective effects on DKD (6). The NLRP3 inflammasome is a critical regulator of inflammation in DKD and is considered a potential therapeutic target (7). In this review, we highlight the role of NLRP3 inflammasome in DKD, and the pathways by which natural products fight against DKD *via* inhibiting the NLRP3 inflammasome activation, so as to offer novel insights for the treatment of DKD.

## 2 A brief overview of the NLRP3 inflammasome in DKD

As a member of NLR, NLRP3 can assemble into the NLRP3 inflammasome after recognizing danger signals, and exert biological effects by activating caspase-1 and promoting the maturation and secretion of interleukin (IL)-1β and IL-18. The NLRP3 inflammasome is a multiprotein complex composed of NLRP3, apoptosis-associated speck-like protein (ASC), and caspase-1. Among them, NLRP3 is the core protein, containing three different domains. First is the pyrin domain (PYD) or C-terminal caspase-recruitment domain (CARD), located on the N-terminus. It can bind to other proteins, and mediate signal transduction. Second is the NACHT domain located in the middle, responsible for activating the NLRP3 inflammasome through ATP-dependent oligomerization. Third is the leucine-rich repeat (LRR) domain, which located on the C-terminus, and is responsible for identifying pathogenic organisms and endogenous danger signals. ASC is an adaptor protein that connects upstream NLRP3 to downstream caspase-1. Caspase-1 is the effector protein in NLRP3 inflammasome, it induces the production and IL-1β and IL-18, resulting in inflammation (8).

## 3 The NLRP3 inflammasome regulator in DKD

NLRP3 is widely expressed in glomerular and tubular epithelial cells of DKD patients and mice. Inhibition of NLRP3, ASC, or caspase-1 can reduce the damage of podocytes, endothelial cells and mesangial cells, and can also significantly reduce the inflammatory response of tubulointerstitium. NLRP3 knockout improved renal pathological changes in diabetic mice (4, 9, 10). These findings suggest that the NLRP3 inflammasome plays a significant role in DKD.

The activation of the NLRP3 inflammasome is a two-step process. The “priming step” is the first phase, involving recognition of danger signals by TLR and activation of NF-κB, which will up-regulate the expression of NLRP3, pro-IL-1β and pro-IL-18. The “activating step” is the second phase, triggered by potassium efflux, calcium influx, mitochondrial dysfunction, lysosomal disruption and reactive oxygen species (ROS) overproduction. These danger signals promote the formation of the NLRP3 inflammasome (11–13). It is found that multiple signaling pathways can exacerbate DKD by targeting the NLRP3 inflammasome activation (**Table 1**).

**Table 1:** Signaling pathways regulating the NLRP3 inflammasome activation in DKD.

Signaling Pathways	*In vivo/in vitro*	Model	Findings	References
NF-κB	*In vitro*	Mouse podocytes	TLR4 knockdown inhibited NLRP3 inflammasome	(14)
	*In vivo, in vitro*	Db/db mice mouse mesangial cell	TLR9 knockdown inhibited NF-κB and NLRP3 inflammasome	(15)
	*In vivo, in vitro*	DKD Patients HFD/STZ mice MPC5	FOXM1 activated SIRT4, inhibited NF-κB and NLRP3 inflammasome	(16)
	*In vivo, in vitro*	Db/db mice STZmice DKD Patients HK-2 cells	CXCL1/CXCR2 activated NF-κB and the NLRP3 inflammasome	(17)
TXNIP	*In vivo, in vitro*	STZ rats HK-2 cells	TXNIP promoted NLRP3 inflammasome activation	(18)
	*In vivo, in vitro*	DKD Patients STZ mice human podocyte cell line	TXNIP activated NADPH oxidase and then triggered NLRP3 inflammasome activation	(19)
	*In vivo, in vitro*	STZ rats Rat glomerular mesangial cells	ROS inhibitor down-regulated TXNIP, NLRP3 and IL-1β	(20)
	*In vivo, in vitro*	db/db mice HK-2 cells	mtROS upregulated TXNIP, NLRP3 and IL-1β	(21)
	*In vivo*	HFD/STZ Rats	IRE1stimulated TXINP and NLRP3	(22)
	*In vivo, in vitro*	STZ mice Human podocyte cell line	EZH2/EGR1/TXNIP/NLRP3 pathway contributed to DKD	(23)
	*In vivo, in vitro*	Db/db mice NRK52E cell line	Sphingosine kinase 2 activated TXINP, NLRP3, and IL-1β	(24)
	*In vitro*	Human glomerular podocytes	NOX4 upregulated NLRP3	(25)
	*In vivo, in vitro*	STZ rats Mouse podocytes	Icariin inhibited NLRP3 by Keap1-Nrf2/HO-1 pathway	(26)
	*In vivo*	Db/db mice	Minocycline stabilized Nrf2 and inhibited NLRP3	(27)
	*In vivo*	STZ mice	Berberine stabilized Nrf2 and inhibited NLRP3	(28)
	*In vivo*	STZ rats	Zinc Oxide Regulated Nrf2/TXNIP/NLRP3 Inflammasome pathway	(29)
	*In vivo, in vitro*	db/db mice HK-2 cells	CD36 promoted mtROS and NLRP3	(30)
	*In vivo, in vitro*	STZ mice Mouse proximal tubular cells	Activated Protein C inhibited ROS and NLRP3	(31)
	*In vitro*	Mouse glomerular mesangial cells	RIPK2 inhibited ROS and NLRP3 inflammasome	(32)
	*In vivo, in vitro*	DKD patients Murine renal tubular epithelial cells	Optineurin reduced mtROS and inhibited NLRP3 inflammasome	(33)
Non-coding RNAs	*In vivo, in vitro*	DKD patients STZ mice& db/db mice Human podocytes	MiRNA-10 negatively regulated NLRP3	(34)
	*In vitro*	Mouse glomerular podocyte line	MiRNA-29a inhibited NLRP3	(35)
	*In vivo, in vitro*	DKD Patients HK-2 cells	MiR-520c-3p inhibited TXNIP/NLRP3	(36)
	*In vivo, in vitro*	STZ rats HK-2 cells	IncRNA-MALAT1 down-regulated miR-23c and up-regulated NLRP3	(37)
	*In vitro*	MPC-5 cells	Atorvastatin protected podocytes by regulating MALAT1/miR-200c/Nrf2	(38)
	*Vitro*	HK-2 cells	IncRNA-MALAT1 down-regulated miR-30c and up-regulated NLRP3	(39)
	*In vivo, in vitro*	STZ rats HBZY-1 cells	IncRNA-NEAT1 down-regulated miR-34c and up-regulated NLRP3	(40)
	*In vitro*	HK-2 cells	IncRNA-NEAT2 down-regulated miR-206 and up-regulated NLRP3	(41)
	*In vivo, in vitro*	STZ rats HK-2 cells	IncRNA-XIST down-regulated miRNA-15b-5p, upregulated TLR4 and NLRP3	(42)
	*In vivo, in vitro*	DKD patients HK-2 cells	IncRNA-KCNQ1OT1 down-regulated miRNA-506-3p and up-regulated NLRP3	(43)
	*In vitro*	HK-2 cells	lncRNA-GAS5 down-regulated miR-452-5p and NLRP3	(44)
	*In vitro*	Mouse podocyte cell line	sC5b-9 promoted NLRP3 activation *via* up-regulating Kcnq1to1and down-regulating mRNA-486a-3p	(45)
	*In vivo, in vitro*	DKD patients HK-2 cells	lncRNA-ANRIL down-regulated miRNA-497, and up-regulated TXNIP/NLRP3	(46)
	*In vitro*	Mouse mesangial cells	IncRNA-Gm4419 knockdown inhibited NF-κB and NLRP3	(47)
	*In vivo, in vitro*	DKD patients HK-2 cells	Circ_0004951 down-regulated miRNA-93-5p, and up-regulated NLRP3	(48)
Interleukin	*In vivo*	Db/db mice	Blockade of IL-6 receptor inhibited NLRP3 by regulating IL-17A	(49)
	*In vivo, in vitro*	DKD patients STZ mice HEK293T cells	IL-22 inhibited NLRP3/caspase-1/IL-1β	(50)
	*In vitro*	Mouse Podocyte	IL-37 inhibited NLRP3	(51)
BTK	*In vivo*	DKD patients STZ mice	BTK activated NLRP3 inflammasome	(52)
	*In vivo, in vitro*	HFD mice Murine bone marrow-derived macrophages Human monocyte-derived macrophages	BTK inhibitor suppressed NLRP3 *via* regulating IRS-1/Akt/GSK-3β	(53)
GSK-3β/HIF-1α	*In vitro*	Mouse renal proximal tubular epithelial cells	GSK-3β knockdown decreased NLRP3	(54)
HDAC6	*In vivo, in vitro*	DKD patients STZ mice Mouse Bone Marrow–Derived Macrophages HK-2 cells	HDAC6 inhibitor suppressed NLRP3 inflammasome	(55)
RIPK3	*In vivo*	STZ mice	RIPK3 controls cellular signaling through the formation of NLRP3	(56)
Syk/JNK	*In vivo, in vitro*	STZ rats HK-2 cells Rat glomerular mesangial cells	Syk/JNK activated NLRP3	(57)
PPARs	*In vivo*	Sugar-induced mice with diabesity	PPAR-δ agonists inhibited NLRP3	(58)
RAC1	*In vivo, in vitro*	Db/db mice HEK293T cells	RAC1 binding to NLRP3 activates the NLRP3 inflammasome	(59)
Spop	*In vivo, in vitro*	STZ mice Mice podocytes	Spop promoted NLRP3 degradation	(60)
WTAP	*In vivo, in vitro*	DKD patients HK-2 cells	WTAP upregulated NLRP3	(61)

CXCL1, Chemokine (C-X-C motif) Ligand 1 Protein; CXCR2, CXC chemokine receptor 2; EGR1, Early Growth Response Protein 1; EZH2, Enhancer of zeste homolog 2; FOXM1, Forkhead box M1; GSK-3β, Glycogen synthase kinase-3β; HDAC6, Histone deacetylase 6; HFD, High-Fat Diet; HIF, Hypoxia inducible factor; IRE1, Inositol-requiring enzyme 1; JNK, c-Jun N-terminal kinase; MPC, mouse podocyte cell; NLRP3, The nucleotide-binding oligomerization domain-like receptor family pyrin domain containing 3; NOX4, Nicotinamide Adenine Dinucleotide Phosphate Oxidase 4; PPARs, Peroxisome-proliferator activated receptors; RAC1,Ras-related C3 botulinum toxin substrate 1; RIPK, Receptor interacting protein kinase; ROS, reactive oxygen species; SIRT4, sirtuin 4; STZ, Streptozocin; Spop, Speckle-type POZ protein; Syk, Spleen tyrosine kinase; TLR, Toll-like receptors; TXNIP, thioredoxin-interacting protein; WTAP, Wilms tumor 1-associated protein.

### 3.1 NF-κB/NLRP3 signaling pathway

NF-κB is a transcription factor in the form of p50/p65 heterodimer. Normally, NF-κB binds to its inhibitor kappa B (IκB) and becomes inactive. When stimulus signals activate IκB kinase, IκB-α is phosphorylated and degraded, allowing NF-κB and IκB are dissociated and translocated into nucleus, where they regulate the expression of target genes (62). Liu et al. (14) revealed that high glucose (HG) promoted the activation of NLRP3 inflammasome in mouse podocytes (MPCs). The expression of TLR4 was also upregulated, which is an important signaling molecule regulating NF-κB. TLR4 knockdown inhibited the activation of NLRP3 inflammasome, attenuated HG-induced cell apoptosis, and increased cell viability. Shen et al (15) found that TLR9 knockdown would inhibit NF-κB/NLRP3 pathway in HG-induced Mesangial Cells (MCs). Furthermore, inhibition of TLR9 reduced NF-κB and NLRP3 expression, and decreased microalbuminuria, renal inflammatory response, and glomerular lesion in db/db mice. Xu et al (16) demonstrated that Forkhead box M1(FOXM1) transcriptionally activated sirtuin 4 (SIRT4) and inhibited NF-κB signaling and the NLRP3 inflammasome, thereby alleviating renal injury *in vivo* and *in vitro*. Tang et al. (17) also confirmed that CXCL1/CXCR2 may cause inflammation in HK-2 cells with HG treatment by phosphorylating NF-κB and activating the NLRP3 inflammasome. Li et al. (63) found that the activation of AMPK/SIRT1 pathway promoted the expression of NF-κB, NLRP3, ASC, Caspase-1, and IL-1β in DKD mice.

### 3.2 TXNIP/NLRP3 signaling pathway

Thioredoxin-interacting protein (TXNIP) is an alpha-arrestin protein with a molecular weight of 46 kD. It can bind to thioredoxin (TRX) and interfere with its expression, which is an essential regulator of oxidative stress, cell proliferation, and apoptosis (64, 65). It has been revealed that TXNIP is released from oxidized TRX under oxidative stress, resulting in the activation of NLRP3 inflammasome (66).

There is abundant evidence that TXNIP/NLRP3 signaling pathway is involved in the inflammatory response of DKD. For instance, Gu et al. (18) revealed that TXNIP and NLRP3 were overexpressed in the renal tissue of DKD rats. It is also observed that HG stimulated TXNIP/NLRP3, promoting inflammation. Gao et al. (19) found that HG-promoted Nicotinamide Adenine Dinucleotide Phosphate (NADPH) oxidase activation *via* TXNIP, which in turn activated the NLRP3 inflammasome, leading to podocyte injury. It has also been revealed that HG activates ROS/TXNIP/NLRP3 inflammasome signaling in glomerular mesangial cells (20), and mitochondrial ROS(mt ROS)/TXNIP/NLRP3 pathway is involved in tubular oxidative injury in DKD (21). Furthermore, it has been confirmed that many molecules participate in the pathogenesis of DKD by regulating TXNIP/NLRP3 inflammasome pathway. Inositol-requiring enzyme 1α (IRE1α), an endoplasmic reticulum transmembrane sensor, can stimulate TXINP/NLRP3 signaling pathway and aggravate DKD in rat model (22). Enhancer of zeste homolog 2 (EZH2), a subunit of the polycomb repressive complex 2, contributes to S-adenosylhomocysteine inhibition-aggravated DKD in mice through EZH2/EGR1/TXNIP/NLRP3 signaling pathway (23). Sphingosine kinase 2 (SphK2) is a key enzyme catalyzing the formation of sphingosine-1-phosphate. Research shows that SphK2 increases TXNIP, NLRP3 inflammasome and IL-1β levels, induces inflammation, promotes renal tubular epithelial cell damage, leading to DKD aggravation (24).

Additionally, there is substantial evidence that many molecules contribute to DKD progression through the ROS/NLRP3 inflammasome pathway. It is found that NADPH oxidase 4 (NOX4), a major source of ROS, is upregulated in HG-induced podocytes. Suppression of NOX4 inhibits the activation of NLRP3 inflammasome and alleviates podocytes apoptosis (25). Nuclear factor E2-related factor 2 (Nrf2) is a transcription factor that protects cells from oxidative stress (67) and serves as most sensitive signal of scavenging ROS under oxidative stress (68). It is reported that Nrf2 may alleviate DKD by suppressing the activation of NLRP3 inflammasome (26–29). CD36, a fatty acid transporter, causes renal tubular epithelial cell injury by activating mtROS/NLRP3 pathway in DKD (30). Activated protein C (aPC), an endothelial-dependent cytoprotective coagulation protease, meliorates tubular mitochondrial ROS and inflammation in DKD (31). Receptor interacting protein kinase 2 (RIPK2) has also been confirmed to negatively regulate ROS/NLRP3 signaling in mouse glomerular mesangial cells treated with HG (32). Optineurin, a well-recognized autophagy receptor, reduces the activation of NLRP3 inflammasome by reducing mtROS and mitophagy in HG -treated renal tubular cells (33).

### 3.3 Non-coding RNAs

Non-coding RNAs (ncRNAs) are recognized as a class of ribonucleic acids (RNAs) that are not translated into proteins. ncRNAs consist of various family members, including microRNAs (miRNAs), long ncRNAs (lncRNAs), ribosomal RNAs, transfer RNAs, circular RNAs(circ-RNAs), and others. Different classes of ncRNAs engage in different cellular processes, regulating gene expression, RNA maturation, and protein synthesis (69).

MiRNAs are small ncRNAs that regulate gene expression through recognizing cognate sequences and interfering with transcriptional, translational, and epigenetic processes. Many miRNAs have been shown to participate in the pathogenesis of DKD by regulating the NLRP3 inflammasome. For example, Ding et al (34) found that miRNA-10 alleviated inflammation in DKD by reducing the NLRP3 inflammasome activation. Zhang (35) demonstrated that miRNA-29a inhibited HG-induced podocytes pyroptosis and alleviated inflammatory response by directly targeting NLRP3. Song et al (36) revealed that miR−520c−3p reduced HK-2 cell pyroptosis induced by HG through inhibiting TXNIP/NLRP3 inflammasome pathway.

LncRNAs are defined as ncRNAs containing more than 200 nucleotides in length (70). They act through numerous paradigms and are key regulatory molecules in cells (71). It is found that in diabetic rats and HG treated podocytes/renal tubule (HK-2) cells, the up-regulation of lncRNA-MALAT1 promoted the NLRP3 inflammasome activation *via* inhibiting miR-23c (37), miR-200c (38) and miR-30c (39). In addition, many other lncRNAs have also been confirmed to take part in the development of DKD by targeting NLRP3 inflammasome, such as IncRNA-NEAT1/miR-34c (40), lncRNA NEAT2/miR-206 (41), lncRNA-XIST/miR-15b-5p (42), lncRNA-KCNQ1OT1/miR-506-3p (43), lncRNA-GAS5/miR-452-5p (44), Kcnq1ot1/miR-486a-3p (45), lncRNA-ANRIL/miR-497 (46), and lncRNA-Gm4419 (47).

Circular RNAs(circ-RNAs) are a class of ncRNAs that lack the 5’ or 3’ end. They regulate gene expression by pervading the transcription, the mRNA turnover, and translation. It is showed that Circ_0004951 is significantly up-regulated in DKD, where it can suppress miR-93-5p and activate NLRP3 inflammasome (48).

### 3.4 Others

Interleukin (IL): ILs are a type of cytokine released by various cells, playing a crucial role in immune regulation and homeostasis. Many ILs have been confirmed to be involved in renal damage caused by diabetes through the regulation of the NLRP3 inflammasome. Wu et al. (49) found that blocking the IL-6 receptor inhibited the NLRP3 inflammasome by restraining IL-17A. Wang et al. (50) demonstrated that IL-22 has reno-protective effects on DKD by downregulating renal NLRP3/caspase-1/IL-1β pathway. Zhang et al. (51) found that IL-37 decreased the expression of NLRP3, ASC, and caspase-1 in HG-treated podocytes.

Bruton’s tyrosine kinase (BTK): BTK, an intracellular non-receptor tyrosine kinase, is considered as an vital signal in immunoregulation (72). It has been observed that BTK activates the NLRP3 inflammasome and promotes renal inflammation in diabetic patients and mice (52). BTK inhibitor attenuates NLRP3 inflammasome activation and alleviates DKD (53).

Glycogen synthase kinase (GSK)-3β/Hypoxia inducible factor(HIF)-α: GSK-3β, a serine/threonine kinase, is crucial for glycogen synthesis by regulating phosphorylation of glycogen synthase (73). It is found that HIF-1α is also a direct target of GSK-3β (74). Inhibition of the GSK-3β/HIF-1α pathway has been shown to alleviate NLRP3-induced pyroptosis in HG-treated renal tubular epithelial cells (54).

Histone deacetylase 6 (HDAC6):HDAC6 is a cytoplasmic enzyme that participates in a variety of cellular processes (75). Inhibition of HDAC6 has been shown to ameliorate DKD by suppressing the NLRP3 inflammasome (55).

Receptor-interacting protein kinase-3 (RIPK3): RIPK3 is a multifunctional regulator of cell death and inflammation. It is reported that RIPK3 is associated with renal fibrosis in DKD by activating NLRP3 inflammasome. Blockade of RIPK3 attenuates tubulointerstitial fibrosis (56).

Spleen tyrosine kinase (Syk)/c-Jun N-terminal kinase (JNK)/NLRP3 signaling pathway: Syk is a non-receptor protein tyrosine kinase. Inhibition of Syk has been shown to downregulate JNK expression and suppress the activation of the NLRP3 inflammasome stimulated by HG, indicating that the Syk/JNK/NLRP3 pathway may play a role in the inflammatory injury in DKD (57).

Peroxisome-proliferator activated receptors (PPARs): PPARs belong to the nuclear receptor superfamily, with three subtypes: PPAR-α, PPAR-γ, and PPARβ/δ. They regulate glucose and lipid metabolism and also mediate inflammation (76). It is reported that PPAR-δ agonist attenuates renal dysfunction and inflammation by preventing activation of the NLRP3 inflammasome in diabesity mice (58).

Ras-related C3 botulinum toxin substrate 1 (RAC1): RAC1 is a member of the Rho family of small GTPases and plays a role in cell proliferation, apoptosis, and inflammation (77, 78). It is revealed that RAC1 binding to NLRP3 activates the NLRP3 inflammasome in the kidney and accelerates DKD pathological processes (59).

Speckle-type POZ protein (Spop): Spop, an E3 ubiquitin ligase, is involved in many cellular processes by promoting the degradation of its target proteins (79, 80). It is observed that Spop inhibits the NLRP3 inflammasome and ameliorates DKD, the possible mechanism is that Spop may directly contact with NLRP3 and promote NLRP3 degradation *via* elevating K48-linked polyubiquitination of NLRP3 (60).

Wilms tumor 1-associated protein (WTAP): WTAP is a critical constituent of the classical m6A methyltransferase, which may cause modification of NLRP3. It has been demonstrated that WTAP upregulates the expression of NLRP3 by increasing the m6A methylation of NLRP3 mRNA, leading to inflammatory response (61).

## 4 Natural products alleviating DKD *via* targeting the NLRP3 inflammasome

**Figure 1:**
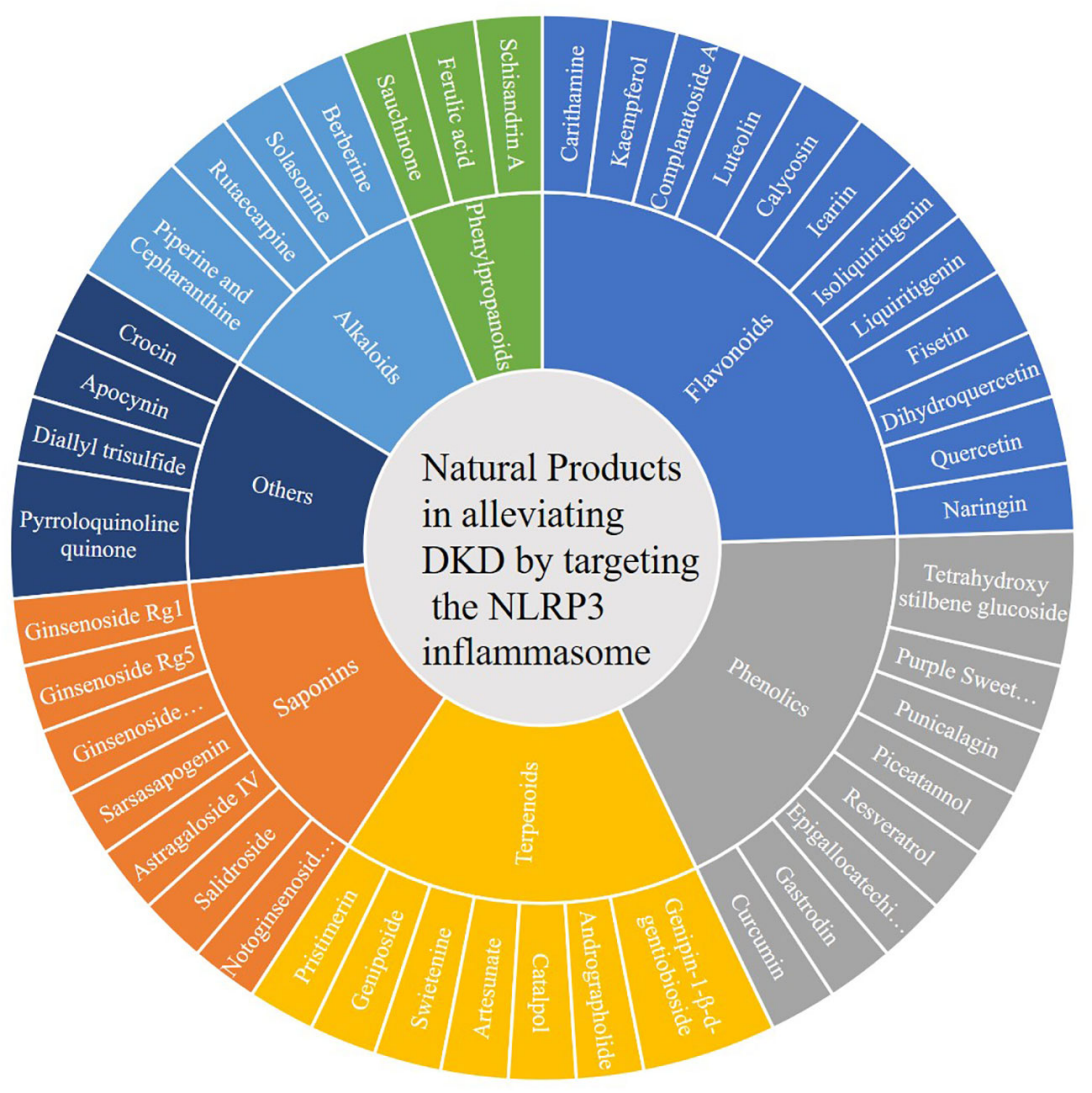
Natural products in alleviating DKD by targeting the NLRP3 inflammasome.

**Figure 2:**
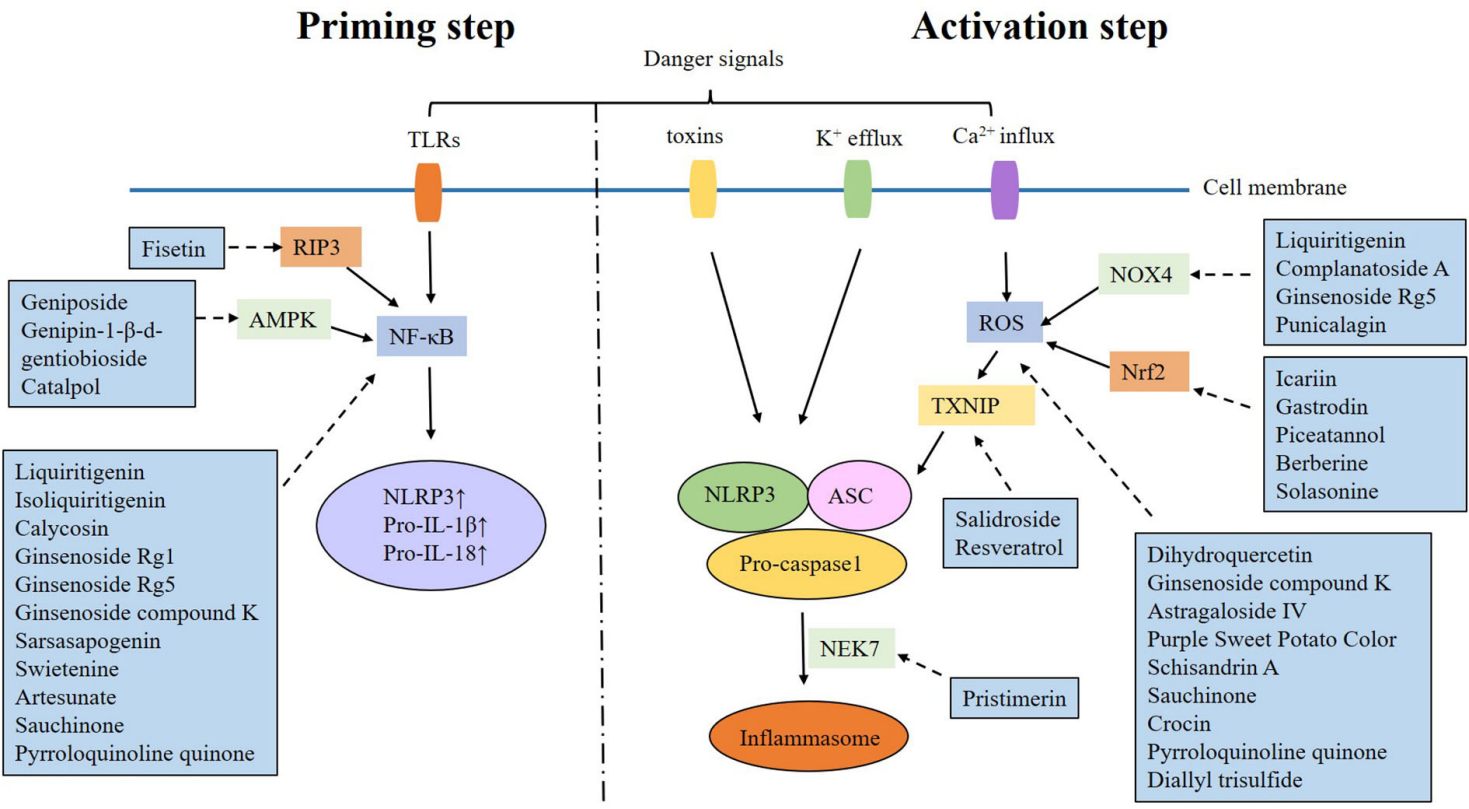
Mechanism of natural products alleviating DKD by targeting NLRP3 inflammasome.

Natural products are chemicals extracted from living organisms, and usually have pharmacological or biological activities. They are highly beneficial for drug design and development. Numerous natural products have been found to alleviate DKD by targeting the NLRP3 inflammasome (**Table 2** and **Figures 1**, **2**).

**Table 2:** Natural products in alleviating DKD by targeting the NLRP3 inflammasome.

	Compounds	Resource	*In Vivo/in Vitro*	Model	Signaling Pathways	References
Flavonoids	Naringin	Grapefruit and citrus fruit	*In vitro*	Glomerular mesangial cells	InhibitedNLRP3	(81)
	Quercetin	Apples, grapes, tomatoes, and onions	*In vivo*	STZ rats	InhibitedNLRP3	(82)
	Dihydroquercetin	Larix sibirica Ledeb. and Pseudotsuga taxifolia (Lamb.) Britton	*In vivo, in vitro*	HFD/STZ rats HBZY-1 and HK2 cell cells	InhibitedROS and NLRP3	(83)
	Fisetin	Vegetables and fruits, apples, persimmons, grapes, strawberries, cucumbers, and onions.	*In vivo, in vitro*	STZ mice Mouse podocytes	Inhibited NLRP3 inflammasome	(84)
	Fisetin	As above	*In vivo, in vitro*	HFD mice HK-2 cells	InhibitedRIP3/NLRP3	(85)
	Liquiritigenin	Glycyrrhizae radix	*In vitro*	HBZY-1	Decreased NOX4, NF-κB and NLRP3	(86)
	Isoliquiritigenin	As above	*In vivo*	STZ rats	Up-regulated Sirt-1 and inhibited NF-κB/NLRP3	(87)
	Icariin	Herba epimedii	*In vivo, in vitro*	STZ rats MPC-5	Inhibited NLRP3 *via* Keap1-Nrf2/HO-1 axis	(26)
	Calycosin	Radix Astragali	*In vivo*	STZ rats	InhibitedNF-κB/p65/NLRP3/TXNIP	(88)
	Luteolin	Fruits and vegetables	*In vitro*	MPC-5	InhibitedNLRP3	(89)
	Complanatoside A	Semen Astragali Complanati	*In vivo, in vitro*	STZ mice HK-2 cells	InhibitedNOX4 and NLRP3	(90)
	Kaempferol	Sand ginger	*In vivo*	STZ rats	InhibitedNLRP3	(91)
	Carithamine	Safflower	*In vivo*	STZ rats	Down-regulated NLRP3	(92)
Saponins	Ginsenoside Rg1	Ginseng	*In vivo, in vitro*	STZ rats Mouse podocyte cell line BNCC337685	Inhibited mTOR/NF-κB/NLRP3	(93)
	Ginsenoside Rg5	Black ginseng	*In vivo*	STZ mice	InhibitedROS, Nox4, TXNIP, NF-κB, MAPK, and NLRP3	(94)
	Ginsenoside compound K	Diol-type ginsenosides	*In vivo, in vitro*	STZ mice HBZY-1	InhibitedROS/NLRP3 and NF-κB/p38	(95)
	Sarsasapogenin	Anemarrhena asphodeloides Bunge	*In vivo, in vitro*	STZ rats Human mesangial cells	Suppressed NLRP3 and NF-κB by down-regulating PAR-1	(96)
	Sarsasapogenin	As above	*In vivo*	STZ rats	InhibitedNLRP3	(97)
	Astragaloside IV	Astragalus membranaceus	*In vivo, in vitro*	Db/db mice Mouse podocytes	InhibitedNLRP3	(98)
	Astragaloside IV	As above	*In vitro*	Mouse mesangial cells(SV40)	Inhibited ROS and NLRP3	(99)
	Salidroside	Rhodiola rosea	*In vitro*	HBZY-1	InhibitedTXNIP-NLRP3	(100)
	Notoginsenoside Fc	Panax notoginseng	*In vivo*	Db/db mice	Inhibited NLRP3	(101)
Phenolics	Tetrahydroxy stilbene glucoside	Polygoni Multiflori Radix	*In vitro*	MPC5	Inhibited NLRP3	(102)
	Gastrodin	Gastrodia elata	*In vitro*	MPC-5 cells	Actived AMPK/Nrf2 and inhibited NLRP3	(103)
	Epigallocatechin-3-gallate	Green tea	*In vivo*	HFD/STZ rats	suppressed endoplasmic reticulum stress-mediated NLRP3 inflammasome overactivation	(104)
	Resveratrol	Grape skin and red wine	*In vivo, in vitro*	STZ rats HK-2 cells	Inhibited TXNIP binding to NLRP3	(105)
	Piceatannol	Grapes, sugar cane, white tea, rhubarb, passion fruit and blueberries	*In vitro*	Mouse podocytes	Upregulated Nrf2 and NLRP3	(106)
	Curcumin	Rhizome Curcuma longa- turmeric	*In vivo, in vitro*	Db/db mice HK-2 cells	InhibitedNLRP3	(107)
	Punicalagin	Pomegranate, myrobalan, leaves of yellow wood, and tropical almond	*In vivo*	HFD/STZ mice	Downregulated NOX4, TXNIP, and NLRP3	(108)
	Purple Sweet Potato Color	Ipomoea batatas	*In vivo*	HFD mice	Suppressed VEGFR2/ROS/NLRP3	(109)
	Grape seed proanthocyanidin	Grape seeds	*In vivo*	STZ rats	InhibitedNLRP3	(110)
Terpenoids	Pristimerin	Celastraceae and Hippocrateaceae	*In vivo, in vitro*	HFD Mice Mouse Bone-marrow cells	Disturbed the interaction between NEK7 and NLRP3	(111)
	Geniposide	Gardenia jasminoides Ellis	*In vivo, in vitro*	STZ mice Mouse podocytes	InhibitedAMPK/SIRT1/NF-κB, and NLRP3	(112)
	Genipin-1-β-d-gentiobioside	As above	*In vivo, in vitro*	STZ mice Mouse Podocytes	Inhibited AMPK/SIRT1/NF-κB, and NLRP3	(63)
	Swietenine	Swietenia macrophylla King	*In vivo, in vitro*	Db/db mice Human mesangial cells	InhibitedNF-κB/NLRP3/Caspase-1	(113)
	Artesunate	Artemisia annua	*In vitro*	HBZY-1	InhibitedTLR4/NF-κB/NLRP3	(114)
	Catalpol	Rehmannia glutinosa	*In vivo, in vitro*	STZ mice Mouse podocytes	InhibitedAMPK/SIRT1/NF-κB and NLRP3	(115)
	Andrographolide	Andrographis paniculata	*In vivo, in vitro*	STZ mice HK-2 cells	Inhibited NLRP3	(116)
	Triptolide	Tripterygium wilfordii Hook F	*In vitro*	Mouse podocytes	Inhibited NLRP3	(117)
Alkaloids	Berberine	Coptis and Phellodendron	*In vivo*	HFD/STZ hamsters	Regulated Nrf2/NLRP3 pathway	(28)
	Berberine	As above	*In vivo, in vitro*	STZ rats HK-2 cells	InhibitedNLRP3	(118)
	Piperine and Cepharanthine	Black pepper and Stephania cepharantha Hayata	*In vivo*	STZ Rats	Both decreased p38MAPK, p-JNK, TNF-α, TXNIP,NF-κB and NLRP3	(119)
	Solasonine	Solanummelongena	*In vitro*	MPC-5	Regulated Nrf2/NLRP3	(120)
	Rutaecarpine	Euodia rutaecarpine	*In vivo, in vitro*	Db/db mice MPC-5	Down-regulatedVEGFR2/NLRP3	(121)
Phenylpropanoids	Schisandrin A	Schisandra chinensis	*In vivo, in vitro*	STZ mice Human renal glomerular endothelial cells	Inhibited NLRP3 *via* AdipoR1/AMPK-ROS/NLRP3	(122)
	Ferulic acid	Tomatoes, sweet corn, rice grain, Cimicifuga racemosa, Angelica sinensis, and ligustici chuanxiong rhizome	*In vivo*	STZ mice	Inhibited NLRP3	(123)
	Sauchinone	Saururus chinensis	*In vivo*	Human renal mesangial cells	Inhibited NF-κB, ROS, and NLRP3	(124)
Others	Crocin	Saffron	*In vivo*	STZ rats	InhibitedROS and NLRP3	(125)
	Pyrroloquinoline quinone	Fruits, vegetables, Gram-negative bacteria, and human breast milk	*In vivo, in vitro*	STZ mice HK-2 cells	ReducedROS and inhibited NF-κB/NLRP3	(126)
	Apocynin	Picrorhiza kurroa	*In vivo*	STZ Rats	InhibitedNLRP3/XIAP	(127)
	Diallyl trisulfide	garlic	*In vivo*	STZ rats	InhibitedROS/NLRP3/Caspase-1	(128)

HFD, high-fat diet; MPC-5, mouse podocyte cell-5; NEK7, never in mitosis A-related kinase 7; PAR-1, protease-activated receptor 1; STZ, streptozotocin; XIAP, X-linked inhibitor of apoptosis protein.

### 4.1 Flavonoids

Flavonoids refer to a series of compounds formed by two benzene rings connected to each other by three carbon atoms, that is, with a C_6_-C_3_-C_6_ structure (129). Natural flavonoids are classified based on their basic structure into flavones, flavanones, isoflavones, flavonols, anthocyanins, and flavan-3-ols. They are widely found in plants.

Naringin is a bioflavonoid mainly found in the fruits of Citrus paradisi Macfadyen, grapefruit, tangerine, and oranges. It appears to have antioxidant, anticancer, and anti-atherosclerosis properties. It is reported that naringin could lower glucose levels (130–132). In rat glomerular mesangial cells induced by HG, the expressions of NLRP3 were significantly higher. Pre-treatment with naringin alleviated the activation of NLRP3 inflammasome, and inhibited cell proliferation (81).

Quercetin (Qu) is a plant flavonoid widely exist in apples, grapes, tomatoes, and onions, etc. Its structure contains phenolic hydroxyl groups and double bonds, which provide strong antioxidant activity (133). Dihydroquercetin (DHQ), also known as taxifolin, is the reduced form of Qu. It is a major dihydroflavone compound derived from Larix sibirica Ledeb. and Pseudotsuga taxifolia (Lamb.) Britton (134). These two natural compounds exert numerous biological activities, including antioxidant, anti-inflammation, antitumor, antiviral effects (135–139). Wang et al. (82) found that Qu can suppress the NLRP3 inflammasome activation in kidney, ameliorating kidney lipid accumulation in STZ-treated rats. A meta-analysis of rodent data (140) also showed that Qu significantly improved renal function, urinary protein excretion, and renal pathological changes in DKD. Regarding the underlying mechanisms, Qu may provide renoprotection in DKD through the mtROS-TRX/TXNIP/NLRP3/IL-1β pathways. For DHQ, it has been shown to significantly reduce microalbuminuria, improve glucose and lipid metabolism dysfunction, and alleviate renal pathological changes in DKD rats. In renal cells induced by HG, DHQ significantly inhibits the activation of NLRP3 inflammasomes and renal fibrosis-associated proteins, reducing cell proliferation and oxidative stress (83).

Fisetin is a natural flavonol extracted from many fruits and vegetables, such as strawberries, apples, cucumbers, and onions (84). Studies indicate that fisetin possesses anti-inflammatory, anti-oxidant, anti-tumor, and cardiovascular protective effect (141–143). Dong et al. (84) demonstrated that fisetin ameliorated podocyte injury caused by HG, and mitigated renal injury in diabetic mice by suppressing NLRP3 inflammasome. Ge et al. (85) found that fisetin significantly attenuated the kidney damage in DKD mice, accompanied by a noticeable reduction in NLRP3 expression in the kidney. The protective effects of fisetin against DKD were also confirmed *in vitro* using palmitate-treated HK2 cells.

Liquiritigenin and Isoliquiritigenin (ISLQ) are flavonoid compounds extracted from Glycyrrhiza radix. Convincing evidence has shown that liquiritigenin and ISLQ possess a diversity of biological properties, such as anti-inflammatory, anti-oxidative, anti-hyperlipidemic, anti-tumor, and hepato-protective efficacity (144–148). Zhu et al. (86) found that liquiritigenin inhibited HG-induced extra-cellular matrix accumulation in glomerular mesangial cells. Moreover, liquiritigenin decreased HG-induced oxidative stress and inflammatory response *via* suppressing NF-κB/NLRP3 pathways. Alzahrani et al. (87) found that in DKD rats, ISLQ protected renal function and attenuated inflammation and collagen formation in kidney by restoring the Sirt-1/NF-κB balance, and downregulating NLRP3 expression.

Icariin (ICA) is obtained from Herba epimedii, and exerts quite a few pharmacological effects, such as anti-fibrosis and anti-inflammation (149). Ding et al. (26) confirmed that ICA increases Sesn2-induced mitophagy to inhibit NLRP3 inflammasome activation by the Keap1-Nrf2/HO-1 signaling pathway in DKD rats.

Calycosin is a representative isoflavone extracted from Radix Astragali (150). Many animal models have demonstrated that calycosin has reno-protective property (151). In diabetic SD rats, calycosin improves the deteriorated kidney functions and proteinuria. The possible mechanism is by regulating NF-κB/p65/NLRP3/TXNIP pathway (88).

Luteolin is a natural flavonoid present in several fruits and vegetables. It possesses many pharmacological properties, such as anti-inflammatory, antioxidant, anti-apoptotic, and anti-cancer effects (152–154). Yu et al. (89) revealed that luteolin could reduce cell apoptotic in HG-treated podocyte, and significantly inhibit the NLRP3 inflammasome activation and IL-1β production in HG-treated MPC-5 cells, suggesting that the anti-apoptotic effect was mostly related to NLRP3 inflammasome.

Complanatoside A (CA) is the ethanolic extract of Semen Astragali Complanati. It exhibits several biological activities, such as anti-oxidant and anti-apoptosis (155), and is widely used to fight against renal diseases in China. Ren et al. (90) found that CA mitigated the pathological lesions of glomeruli and tubular interstitium in DKD mice, it also reduced epithelial-mesenchymal transition (EMT) of HK-2 cells *via* blocking NOX4 expression and NLRP3 inflammasome activation.

Kaempferol is a natural compound with the formula C_15_H_10_O_6_. It is mainly derived from the roots and stems of sand ginger, and is also distributed in plants such as tea, broccoli, and grapefruit. It is reported that kaempferol has antibacterial, anti-inflammatory, antioxidant, antitumor and many other pharmacological effects (156, 157). Studies revealed that kaempferol improved proteinuria and renal function in DKD rats. It also relieved renal tissue damage and cell apoptosis. Since the expression of NLRP3, ASC, and caspase-1 was decreased, it is probable that kaempferol can alleviate kidney damage in DKD rats by inhibiting the NLRP3 inflammasome (91).

Carithamine is a natural flavochrome extracted from the petals of safflower that has a variety of pharmacological properties, such as dilating coronary arteries, protecting myocardium and brain tissue, antioxidant, and immunoregulation (158). Gao et al. (92) found that intraperitoneal injection of carithamine alleviated proteinuria in DKD rats by downregulating the expression of NLRP3 and Caspase-1.

### 4.2 Saponins

Saponins are a class of glycosides whose aglycones are triterpenoids or spirostanes. They are mainly found in terrestrial higher plants but can also be found in marine organisms such as starfish and sea cucumbers (159). Saponins are generally considered beneficial for the cardiovascular system and diabetes (160).

Ginsenoside is the major active constituent of ginseng, which belongs to perennial herbaceous plant and is used as a traditional herb medicine for many years (161). Ginsenoside owns many biological activities, including anti-inflammation, anti-tumor, and anti-diabetes (162–164). Ginsenoside Rg1 and Ginsenoside Rg5 are the representative monomers of ginsenoside (165, 166). Wang et al. (93) found that ginsenoside Rg1 inhibited pyroptosis in hyperlipid-induced podocytes, and this effect was also observed in the kidneys of rats with DKD. The possible mechanism was by down-regulating the mTOR/NF-κB/NLRP3 pathway. Zhu et al. (94) demonstrated that ginsenoside Rg5 reduced oxidative stress and the activation of NLRP3 inflammasome, thereby mitigated kidney damage in DKD mice. Ginsenoside compound K(CK) is the final metabolite of diol-type ginsenosides such as Rb1 and Rb2 by the action of intestinal flora (167). Song et al. (95) proved that CK significantly improved renal function and urinary protein excretion of DKD mice, and the proliferation of glomerular mesangial matrix was also decreased. Moreover, the protective effect of CK is possibly due to suppression of NF-κB/p38 and ROS/NLRP3 signaling pathway.

Sarsasapogenin (Sar) is a steroidal sapogenin isolated from Anemarrhena asphodeloides Bunge. It is believed to have antiplatelet, antithrombotic, and anti-inflammatory propertis (168–171). Tang et al. (96) found that Sar significantly improved kidney function in DKD rats, and renal histopathology showed that it reduced mesangial cell proliferation, inhibited the activation of NLRP3 inflammasome and NF-κB. Liu et al. (97) also found that Sar can markedly ameliorate DKD in rats *via* ameliorating the NLRP3 inflammasome activation and AGEs–receptor for AGE (RAGE) interaction.

Astragaloside IV (AS−IV) is the primary active ingredient in Astragalus membranaceus, a traditional herb medicine. It has been identified to have anti-inflammatory and anti-oxidant effects and is widely used to deal with diabetes and cardiovascular diseases (172, 173). Feng et al. (98) demonstrated that AS-IV blocked NLRP3 inflammasome activation, and improved renal function and podocytes damage in db/db mice, exerting a reno-protective effect. Zhao et al. (99) observed that AS−IV reduced NLRP3 expression in HG exposed mouse glomerular mesangial cells.

Salidroside (SAL) is the predominant component of Rhodiola rosea, an herbal plant with a wide range of pharmacological effects, including anti-altitude sickness, anti-oxidant, and anti-diabetes (174–176). SAL also exerts beneficial effects on DKD (177). Wang et al. (100) showed that SAL inhibited TXNIP/NLRP3 signaling pathway in rat glomerular mesangial cells.

Notoginsenoside Fc (Fc) is a novel saponin extracted from Panax notoginseng with excellent anti-platelet aggregation ability (178). It is reported that Fc reduced albuminuria, alleviated renal failure, and relieved podocyte injury in db/db mice by inhibiting the NLRP3 inflammasomes (101).

### 4.3 Phenolics

Phenolics are a class of chemicals that contain aromatic rings and hydroxyl groups. They are widely distributed in nature, especially in fruits, vegetables, cereals, flowers, spices, and teas (179). In the past decades, the potential value of phenolics in healing DKD has been explored.

Tetrahydroxy stilbene glucoside (TSG) is derived from Polygoni Multiflori Radix. TSG has been shown to reduce blood cholesterol, protect the liver, possess antioxidant abilities, and exhibit anti-atherosclerotic properties (180). Li et al. (102) demonstrated that TSG prevented podocytes apoptosis in HG condition, and it was partly through the blockade of NLRP3 inflammasome.

Gastrodin is a natural compound isolated from the dried root of Gastrodia elata (181). It has been found to exert anti-inflammatory, antioxidative, and neuroprotective effects (182). Huang et al. (103) proved that gastrodin halted the activation of NLRP3 inflammasome in HG-treated podocytes, which reduced renal inflammation and oxidative stress.

Epigallocatechin-3-gallate (EGCG) is a polyphenolic component found in tea leaves with strong anti-inflammatory property. Yang et al. (104) confirmed that EGCG can ameliorate renal dysfunction and renal histopathological injury in DKD rats. Furthermore, the reno-protective effects of EGCG are mainly related to the suppression of endoplasmic reticulum stress-mediated NLRP3 inflammasome overactivation.

Resveratrol is a polyphenolic compound mainly derived from plants such as grapes, peanuts, mulberries, and Polygonum cuspidatum (183). It is reported to be a strong scavenger of ROS (184), and has the ability to ameliorate hyperglycemia mediated renal dysfunction (185). Xiao et al. (105) revealed that in diabetic models with acute kidney injury, the primary mechanism is attributed to TXNIP/NLRP3 activation stimulated by oxidative stress.

Piceatannol is a polyphenol compound sharing a similar chemical structure to resveratrol. It is mainly found in grapes, sugar cane, white tea, rhubarb, passion fruit and blueberries (186). Piceatannol is considered to have anticancer, anti-atherogenic, anti-oxidative, anti-inflammatory, anti-microbial and estrogenic activities, and is widely used in the treatment of heart disease, leukemia and cancer (187–190). Yao et al. found that piceatannol can inhibit apoptosis, inflammation and oxidative stress of podocytes under HG condition. The possible mechanism is that it inhibits the activation of NLRP3 inflammation by promoting Nrf2 nuclear translocation and up-regulating Nrf2 expression (106).

Curcumin, a chief component of Curcuma longa, has been consumed by humans as a spice. It exhibits powerful anti-inflammatory and anti-cancer properties (191). The reno-protective effect of curcumin in DKD rats has been verified (192, 193). Lu et al. (107) found that curcumin inhibited the activation of NLRP3 inflammasome in db/db mice, similar to that in HG-induced HK-2 cells, resulting in alleviation of DKD.

Punicalagin (PU) is the main component of pomegranate polyphenols and is found abundantly in pomegranate, myrobalan, leaves of yellow wood, and tropical almond (194, 195). PU exhibits strong antioxidative, anti-inflammatory, and antineoplastic properties (196, 197). An et al. (108) proved that PU reduced kidney damage in high-fat diet (HFD)/streptozotocin (STZ) mice, possibly by downregulating the NOX4/TXNIP/NLRP3 pathway.

Purple Sweet Potato Color (PSPC) is a natural flavonoid leached from the rhizome of purple sweet potatoes. It has strong anti-oxidant and anti-inflammatory abilities that can protect the brain, liver, and kidney (198–200). Zheng et al. (109) found that PSPC exert renal protection in HFD-treated mice by inhibiting ROS-Triggered NRLP3 inflammation.

Grape seed proanthocyanidin is a polyphenol compound extracted from grape seeds, which is one of the most efficient antioxidants found to date. It has anti-radiation, anti-cancer, anti-atherosclerosis and anti-diabetic effects (201–203). Qiu et al. (110) found that in DKD rats with ischemia-reperfusion injury, intraperitoneal injection of grape seed proanthocyanidin could improve renal function and alleviate renal oxidative stress, possibly by inhibiting NLRP3 gene expression.

### 4.4 Terpenoids

Terpenoids are olefin compounds with an isoprene unit (C_5_ unit) as the basic structural unit. They exist widely in nature and are the main components of some plant fragrances, resins and pigments. Terpenoids have diverse physicochemical properties and biological activities, and exhibit promising efficacy in the management of DKD (204).

Pristimerin (Pri) is a quinonoid triterpene isolated from Celastraceae and Hippocrateaceae (205). It shows excellent anti-bacterial, anti-fungal, anti-inflammatory, and anti-tumor abilities (206, 207), and has been widely used in treating colitis, sepsis, and neuroinflammation (208, 209). Zhao et al. (111) found that intraperitoneal injection of Pri in an HFD-induced diabetic mouse model reversed metabolic disorders by restraining the activation of the NLRP3 inflammasome. They further illustrated that this was associated with disturbing the interaction between never in mitosis A-related kinase 7 (NEK7) and NLRP3 *in vitro*.

Geniposide (GE) and genipin-1-β-d-gentiobioside (GG) are active ingredients extracted from the fruit of Gardenia jasminoides Ellis. Many researches on GE have proved that it can lower blood glucose and lose weight, it also has anti-inflammatory, anti-tumor, neuroprotective, and myocardial protective effects (210–212). Hu et al. (213) revealed that GE can alleviated the development of STZ-induced DKD. Li et al. (112) confirmed that GE down-regulated the expression of NLRP3, ASC, IL-1, and Caspase-1β in DKD mice, possibly through down-regulation of the AMPK/SIRT1/NF-κB signaling pathway. GG has a chemical structure similar to that of GE, except for one more glycosidic group. Li et al. (63) suggested that GG promoted podocyte survival and attenuated renal damage in DKD mice, with the reno-protective effect related to the AMPK/SIRT1/NF-κB/NLRP3 pathway.

Swietenine (Swi) is derived from the Swietenia macrophylla King plant and possesses outstanding anti-bacterial, anti-inflammatory, anti-oxidant, anti-tumor, and anti-diabetic properties (214–216). Duan et al. (113) found that Swi remarkably improved renal function and suppressed inflammatory response in DKD mice. The signal pathway that may be involved is NF-κB/NLRP3/Caspase-1 axis.

Artesunate (ART) is a major derivative of artemisinin isolated from Artemisia annua (217). Studies have revealed that ART possesses a wide range of biological activities, including anti-malarial, anti-oxidative, anti-inflammatory, and anti-tumor effects (218–220). Sun et al. (114) demonstrated that ART inhibited TLR4/NF-κB/NLRP3 pathway, thereby ameliorating glomerular mesangial cell injury under HG conditions.

Catalpol (Cat) is an iridoid glycoside rich in the roots of Rehmannia glutinosa, exhibiting potent anti-oxidant, anti-tumor, anti-inflammatory, and anti-diabetic effects (221, 222). There are accumulating evidence suggesting that Cat can be used to treat DKD (223). Chen et al. (115) revealed that Cat effectively attenuated kidney damage in DKD mice, it can reduce oxide stress and inflammation by targeting AMPK/SIRT1/NF-κB pathway.

Andrographolide is a labdane diterpenoid isolated from Andrographis paniculata Nees with numerous biological activities, including anti-inflammatory, anti-tumor, and anti-diabetic capacities (224). Li et al. (225) found that andrographolide attenuated DKD progression by inhibiting oxidative stress and inflammation in mesangial cells. Moreover, they found that andrographolide significantly reduced HG-induced apoptosis, EMT, and fibrosis *via* blocking NLRP3 inflammasome activation (116).

Triptolide (TP) is the main active ingredient isolated from Tripterygium wilfordii Hook F. It exhibits excellent anti-inflammatory and anti-apoptosis abilities, as well as anti-cancer and anti-diabetic activities (226–228). Wu et al. (117) discovered that TP can block the activation of NLRP3 inflammasome and alleviate EMT in podocytes under HG condition, which may be one of the mechanisms by which TP alleviates podocytes injury in DKD.

### 4.5 Alkaloids

Alkaloids are a class of nitrogen-containing basic organic compounds, which mainly exist in plant. Alkaloids have abundant medicinal value, possessing anti-arrhythmia, anti-hypertensive, analgesic, anti-inflammatory, and anti-cancer properties (229, 230).

Berberine (BBR), also known as berberine hydrochloride or berberine sulphate, is an alkaloid derived from Coptis. It shows anti-inflammatory, anti-oxidant, anti-diabetic, and hypolipidemic activities (231). It is reported that BBR relieves DKD by inhibiting mesangial cell proliferation and ameliorating tubulointerstitial fibrosis (232, 233). Ding et al. (28) revealed that BBR can reduce oxidative stress and antagonize inflammation by regulating Nrf2/NLRP3 pathway. Ma et al. (118) also confirmed that BBR could inhibit HG induced EMT and renal interstitial fibrosis by down-regulating the NLRP3 inflammasome in HK-2 cells.

Piperine (Pip) is a bioactive alkaloid mainly present in black pepper. It has many pharmaceutical effects including promoting digestion, lowering lipid peroxidation, as well as anti-inflammatory, anti-cancer, and antioxidant (234–236). Cepharanthine (CEP) is a natural alkaloid extracted from Stephania cepharantha Hayata, and possesses anti-oxidative, anti-inflammatory, anti-proliferative, anti-metastatic and anti-atherosclerosis properties (237, 238). Samra et al. (119) found that CEP, Pip or their combination noticeably improves renal function and proteinuria in diabetic rats, accompanied by down-regulation of NF-κB and NLRP3.

Solasonine (SS) is a natural glycoalkaloid isolated from Solanummelongena. It has been proved to possess anti‐inflammatory, anti-cancer, and neuroprotective properties (239–241). Zhang et al. (120) revealed that SS alleviated cell apoptosis, reduced pyroptosis and oxidative injury in podocytes induced by HG. The possible mechanism may be through regulating the Nrf2/NLRP3 signaling pathway.

Rutaecarpine is an important active component of Euodia rutaecarpine (242). Numerous studies have shown that rutaecarpine has anti-inflammatory, anti-atherosclerosis, and anti-cancer pharmacological effects (243–245). Hu et al. (121) found that rutaecarpine effectively alleviated renal damage in db/db mice, along with the reduced expression of NLRP3/ASC/IL-18/IL-1β in the kidney. *In vitro* studies also confirmed that rutaecarpine can inhibit NLRP3/ASC/IL-18/IL-1β in MPC-5 and reduce programmed cell necrosis, which suggested that rutaecarpine may be protective to DKD through NLRP3-dependent pathway.

### 4.6 Phenylpropanoids

Phenylpropanoids are one of the main phenolic acids widely distribution in plants, with the C6-C3 carbon skeleton as core structure (246). They are mainly found in fruits, vegetables, cereal grains, beverages, spices and herbs. Phenylpropanoids are known to have multifaceted effects, including antimicrobial, antioxidant, anti-inflammatory, anti-diabetic, anticancer activities (247, 248). Their therapeutic effects on DKD are also being explored.

Schisandra chinensis is the dried ripe fruits of Schisandra chinensis (Turcz.) Baill. It is both a health food and a traditional herb medicine (249, 250).. Schisandrin A is the main lignan derived from Schisandra chinensis, which exerts anti-oxidative, anti-apoptosis, and sedative abilities (251, 252). Wang et al. (122) revealed that schisandrin A decreased ROS overproduction and inhibited inflammation in DKD mice. It also reduced HG-induced ferroptosis and ROS-mediated pyroptosis by mitochondrial damage in human renal glomerular endothelial cells. The expression of TXNIP and NLRP3 was down-regulated by Schisandrin A, suggesting that Schisandrin A attenuated DKD by suppressing TXNIP/NLRP3 signaling pathway.

Ferulic acid (FA) is a natural derivative of caffeic acid commonly found in vegetables, especially in tomatoes, corns, and rices. It is also the main active ingredient of many traditional herbal medicines, involving Cimicifuga racemosa, Angelica sinensis, and ligustici chuanxiong rhizome (253). FA exhibits a wide range of therapeutic effects, including scavenging free radicals, antioxidant properties, and anti-cancer, anti-inflammatory, anti-fibrotic, and reno-protective effects against cardiovascular diseases, neurodegenerative diseases, and diabetes (253–256). It is also revealed that FA has reno-protective effects in DKD rats by antioxidation, anti-inflammation and anti-fibrosis (257–259). Ma et al. (123) further proved that FA reduced the expressions of p62, NLRP3 and IL-1β in renal tissues of DKD mice and suppressed inflammation.

Sauchinone is a biologically active lignin extracted from Saururus chinensis. Studies have shown that it has powerful anti-oxidant, anti-inflammatory, anti-apoptosis, anti-cancer and anti-obesity abilities (260–263). Yoon et al. (124) found that sauchinone improved angiotensin II-induced mesangial inflammation by inhibiting the NLRP3 inflammasome.

### 4.7 Others

Crocin is a carotenoid compound mainly exist in saffron, which belongs to the iris family, a perennial stemless herb. Previous studies have shown that crocin has a variety of pharmacological effects, including the inhibition of cancer growth, inflammatory responses, apoptosis, and oxidative stress (264–268). Crocin also has reno-protective effects (269). Zhang et al. (125) demonstrated that Crocin improved diabetic kidney dysfunction and renal fibrosis in STZ rat. Additionally, Crocin reduced excessive ROS production and decreased the synthesis of pro-inflammatory factors by inhibiting the activation of the NLRP3 inflammasome.

Pyrroloquinoline quinone (PQQ) is the third coenzyme of oxidoreductase discovered so far, which exists widely in plants, bacteria, animals, and human (270). The confirmed biological abilities of PQQ include antioxidant, neuro-protection, and immunoregulation (271–273). Qu et al. (126) demonstrated that PQQ down-regulated the expression of NLRP3, caspase-1, IL-1β, and attenuated renal fibrosis by alleviating mitochondrial dysfunction, reducing ROS production in STZ mice and HG induced HK-2 cells.

Apocynin is a compound isolated from the root of the medicinal herb Picrorhiza kurroa (274, 275). It is used as an antioxidant due to the ability to inhibit NADPH oxidase activity and reduce ROS production (276, 277). Xin et al. (127) found that in rats with DKD, apocynin improved renal function and attenuated renal fibrosis. This effect was likely due to the down-regulation of the NLRP3/X-linked inhibitor of apoptosis protein (XIAP) signaling pathway.

Diallyl trisulfide (DATS), one of the main allyl sulfur compounds exist in garlic, possesses considerable anti-oxidant, anti-inflammatory, anti-fibrosis, and anti-fungal activities (278–280). Shen et al. (128) confirmed that DATS alleviated renal damage in DKD rats, and the expressions of ROS, NLRP3, ASC, Caspase-1, IL-1β and IL-18 were decreased, which suggested that DATS may be effective in treating DKD by inhibiting ROS/NLRP3/Caspase-1 pathway.

## 5 Conclusion

Inflammation plays a crucial role in the pathogenesis of DKD, and the NLRP3 inflammasome is regarded as a key regulator of inflammation in DKD. Various signaling pathways are involved in the activation of NLRP3 inflammasome, including NF-κB, ROS/TXNIP, ncRNAs. However, specific mechanisms and crosstalk between them require further investigation. Many natural products exhibit excellent anti-inflammatory properties, and may alleviate DKD by inhibiting the activation of NLRP3 inflammasome. However, most studies are mainly limited to *in vitro* and animal experiments. With improved understanding of the regulatory network of NLRP3 inflammasome, and better understanding of the pharmacological mechanism of natural products, more clinical trials on the use of natural products in the treatment of DKD are expected in the future.

## Author contributions

All authors contributed significantly to this work and approved the publication of the manuscript. PL devised the research plan. YW, ZS, and MW wrote the manuscript. PL and YW modified and polished the manuscript. All authors contributed to the article and approved the submitted version.

## References

[1] Saran R Robinson B Abbott KC Bragg-Gresham J Chen X Gipson D. US Renal data system 2019 annual data report: epidemiology of kidney disease in the united states. Am J Kidney Dis (2020) 75(1 Suppl 1):A6–7. doi: 10.1053/j.ajkd.2019.09.003 31704083

[2] Niewczas MA Pavkov ME Skupien J Smiles A Md Dom ZI Wilson JM. A signature of circulating inflammatory proteins and development of end-stage renal disease in diabetes. Nat Med (2019) 25(5):805–13. doi: 10.1038/s41591-019-0415-5 PMC6508971 31011203

[3] Ma T Li X Zhu Y Yu S Liu T Zhang X. Excessive activation of notch signaling in macrophages promote kidney inflammation, fibrosis, and necroptosis. Front Immunol (2022) 13:835879. doi: 10.3389/fimmu.2022.835879 35280997 PMC8913942

[4] Tang SCW Yiu WH. Innate immunity in diabetic kidney disease. Nat Rev Nephrol (2020) 16(4):206–22. doi: 10.1038/s41581-019-0234-4 31942046

[5] Rodrigues T Reker D Schneider P Schneider G. Counting on natural products for drug design. Nat Chem (2016) 8(6):531–41. doi: 10.1038/nchem.2479 27219696

[6] Zhou K Zi X Song J Zhao Q Liu J Bao H. Molecular mechanistic pathways targeted by natural compounds in the prevention and treatment of diabetic kidney disease. Molecules (2022) 27(19):6221. doi: 10.3390/molecules27196221 36234757 PMC9571643

[7] Williams BM Cliff CL Lee K Squires PE Hills CE. The role of the NLRP3 inflammasome in mediating glomerular and tubular injury in diabetic nephropathy. Front Physiol (2022) 13:907504. doi: 10.3389/fphys.2022.907504 35755447 PMC9218738

[8] Qiu YY Tang LQ. Roles of the NLRP3 inflammasome in the pathogenesis of diabetic nephropathy. Pharmacol Res (2016) 114:251–64. doi: 10.1016/j.phrs.2016.11.004 27826011

[9] Shahzad K Fatima S Khawaja H Elwakiel A Gadi I Ambreen S. Podocyte-specific Nlrp3 inflammasome activation promotes diabetic kidney disease. Kidney Int (2022) 102(4):766–79. doi: 10.1016/j.kint.2022.06.010 35779608

[10] Wu M Yang Z Zhang C Shi Y Han W Song S. Inhibition of NLRP3 inflammasome ameliorates podocyte damage by suppressing lipid accumulation in diabetic nephropathy. Metabolism (2021) 118:154748. doi: 10.1016/j.metabol.2021.154748 33675822

[11] Lin M Tang SC. Toll-like receptors: sensing and reacting to diabetic injury in the kidney. Nephrol Dial Transplant (2014) 29(4):746–54. doi: 10.1093/ndt/gft446 24203812

[12] Man SM Kanneganti TD. Regulation of inflammasome activation. Immunol Rev (2015) 265(1):6–21. doi: 10.1111/imr.12296 25879280 PMC4400844

[13] Zhang Y Yang W Li W Zhao Y. NLRP3 inflammasome: checkpoint connecting innate and adaptive immunity in autoimmune diseases. Front Immunol (2021) 12:732933. doi: 10.3389/fimmu.2021.732933 34707607 PMC8542789

[14] Liu Y Xu Z Ma F Jia Y Wang G. Knockdown of TLR4 attenuates high glucose-induced podocyte injury *via* the NALP3/ASC/Caspase-1 signaling pathway. BioMed Pharmacother (2018) 107:1393–401. doi: 10.1016/j.biopha.2018.08.134 30257355

[15] Shen J Dai Z Li Y Zhu H Zhao L. TLR9 regulates NLRP3 inflammasome activation *via* the NF-kB signaling pathway in diabetic nephropathy. Diabetol Metab Syndr (2022) 14(1):26. doi: 10.1186/s13098-021-00780-y 35120573 PMC8815223

[16] Xu X Zhang L Hua F Zhang C Zhang C Mi X. FOXM1-activated SIRT4 inhibits NF-kappaB signaling and NLRP3 inflammasome to alleviate kidney injury and podocyte pyroptosis in diabetic nephropathy. Exp Cell Res (2021) 408(2):112863. doi: 10.1016/j.yexcr.2021.112863 34626587

[17] Tang H Yang M Liu Y Liu H Sun L Song P. The CXCL1-CXCR2 axis mediates tubular injury in diabetic nephropathy through the regulation of the inflammatory response. Front Physiol (2021) 12:782677. doi: 10.3389/fphys.2021.782677 34975537 PMC8716832

[18] Gu C Liu S Wang H Dou H. Role of the thioredoxin interacting protein in diabetic nephropathy and the mechanism of regulating NOD−like receptor protein 3 inflammatory corpuscle. Int J Mol Med (2019) 43(6):2440–50. doi: 10.3892/ijmm.2019.4163 PMC6488169 31017263

[19] Gao P He FF Tang H Lei CT Chen S Meng XF. NADPH oxidase-induced NALP3 inflammasome activation is driven by thioredoxin-interacting protein which contributes to podocyte injury in hyperglycemia. J Diabetes Res (2015) 2015:504761. doi: 10.1155/2015/504761 25834832 PMC4365330

[20] Feng H Gu J Gou F Huang W Gao C Chen G. High glucose and lipopolysaccharide prime NLRP3 inflammasome *via* ROS/TXNIP pathway in mesangial cells. J Diabetes Res (2016) 2016:6973175. doi: 10.1155/2016/6973175 26881256 PMC4736396

[21] Han Y Xu X Tang C Gao P Chen X Xiong X. Reactive oxygen species promote tubular injury in diabetic nephropathy: the role of the mitochondrial ros-txnip-nlrp3 biological axis. Redox Biol (2018) 16:32–46. doi: 10.1016/j.redox.2018.02.013 29475133 PMC5842313

[22] Ke R Wang Y Hong S Xiao L. Endoplasmic reticulum stress related factor IRE1alpha regulates TXNIP/NLRP3-mediated pyroptosis in diabetic nephropathy. Exp Cell Res (2020) 396(2):112293. doi: 10.1016/j.yexcr.2020.112293 32950473

[23] Dai X Liao R Liu C Liu S Huang H Liu J. Epigenetic regulation of TXNIP-mediated oxidative stress and NLRP3 inflammasome activation contributes to SAHH inhibition-aggravated diabetic nephropathy. Redox Biol (2021) 45:102033. doi: 10.1016/j.redox.2021.102033 34119876 PMC8209273

[24] Yan S-S. Sphk2 promotes diabetic nephropathy through regulation of renal tubular epithelial cells injury. Guangzhou, Guangdong, China: Guangdong Pharmaceutical University (2019).

[25] Wang B Zheng B Ma P An D Wang R. The role of NOX4 in regulating NALP3 inflammasome expression in high glucose-induced podocyte apoptosis. Henan Med Res (2019) 28(10):1729–33.

[26] Ding X Zhao H Qiao C. Icariin protects podocytes from NLRP3 activation by Sesn2-induced mitophagy through the Keap1-Nrf2/HO-1 axis in diabetic nephropathy. Phytomedicine (2022) 99:154005. doi: 10.1016/j.phymed.2022.154005 35247669

[27] Shahzad K Bock F Al-Dabet MM Gadi I Nazir S Wang H. Stabilization of endogenous Nrf2 by minocycline protects against Nlrp3-inflammasome induced diabetic nephropathy. Sci Rep (2016) 6:34228. doi: 10.1038/srep34228 27721446 PMC5056367

[28] Ding B Geng S Hou X Ma X Xu H Yang F. Berberine reduces renal cell pyroptosis in golden hamsters with diabetic nephropathy through the Nrf2-NLRP3-Caspase-1-GSDMD pathway. Evid Based Complement Alternat Med (2021) 2021:5545193. doi: 10.1155/2021/5545193 35971382 PMC9375700

[29] Abd El-Khalik SR Nasif E Arakeep HM Rabah H. The prospective ameliorative role of zinc oxide nanoparticles in STZ-induced diabetic nephropathy in rats: mechanistic targeting of autophagy and regulating Nrf2/TXNIP/NLRP3 inflammasome signaling. Biol Trace Elem Res (2022) 200(4):1677–87. doi: 10.1007/s12011-021-02773-4 34241775

[30] Hou Y Wang Q Han B Chen Y Qiao X Wang L. CD36 promotes NLRP3 inflammasome activation *via* the mtROS pathway in renal tubular epithelial cells of diabetic kidneys. Cell Death Dis (2021) 12(6):523. doi: 10.1038/s41419-021-03813-6 34021126 PMC8140121

[31] Rana R Manoharan J Gupta A Gupta D Elwakiel A Khawaja H. Activated protein c ameliorates tubular mitochondrial reactive oxygen species and inflammation in diabetic kidney disease. Nutrients (2022) 14(15):3138. doi: 10.3390/nu14153138 35956315 PMC9370435

[32] Gao C Chen J Fan F Long Y Tang S Jiang C. RIPK2-mediated autophagy and negatively regulated ROS-NLRP3 inflammasome signaling in GMCs stimulated with high glucose. Mediators Inflamm (2019) 2019:6207563. doi: 10.1155/2019/6207563 31485193 PMC6710801

[33] Chen K Feng L Hu W Chen J Wang X Wang L. Optineurin inhibits NLRP3 inflammasome activation by enhancing mitophagy of renal tubular cells in diabetic nephropathy. FASEB J (2019) 33(3):4571–85. doi: 10.1096/fj.201801749RRR 30571313

[34] Ding H Li J Li Y Yang M Nie S Zhou M. MicroRNA-10 negatively regulates inflammation in diabetic kidney *via* targeting activation of the NLRP3 inflammasome. Mol Ther (2021) 29(7):2308–20. doi: 10.1016/j.ymthe.2021.03.012 PMC8261077 33744467

[35] Zhang J. The mechanism of miR-29a targeting regulation NLRP3 on glomerular podocyte pyroptosis under high glucose condition and analysis of the influencing factors of death in maintenance hemodialysis patients. Kunming, Yunnan, China: Kunming Medical University (2021).

[36] Song Y Guo F Gan T Shao M Fan X Xu Y. Effect of miR−520c−3p on pyroptosis of proximal tubular epithelial cells in diabetic kidney disease and its mechanism. Chin J Diabetes Mellitus (2022) 14(6):592–602. doi: 10.3760/cma.j.cn115791-20210923-00513

[37] Li X Zeng L Cao C Lu C Lian W Han J. Long noncoding RNA MALAT1 regulates renal tubular epithelial pyroptosis by modulated miR-23c targeting of ELAVL1 in diabetic nephropathy. Exp Cell Res (2017) 350(2):327–35. doi: 10.1016/j.yexcr.2016.12.006 27964927

[38] Zuo Y Chen L He X Ye Z Li L Liu Z. Atorvastatin regulates MALAT1/miR-200c/NRF2 activity to protect against podocyte pyroptosis induced by high glucose. Diabetes Metab Syndr Obes (2021) 14:1631–45. doi: 10.2147/DMSO.S298950 PMC8053520 33880049

[39] Liu C Zhuo H Ye MY Huang GX Fan M Huang XZ. LncRNA MALAT1 promoted high glucose-induced pyroptosis of renal tubular epithelial cell by sponging miR-30c targeting for NLRP3. Kaohsiung J Med Sci (2020) 36(9):682–91. doi: 10.1002/kjm2.12226 PMC11896152 32391974

[40] Zhan JF Huang HW Huang C Hu LL Xu WW. Long non-coding RNA NEAT1 regulates pyroptosis in diabetic nephropathy *via* mediating the miR-34c/NLRP3 axis. Kidney Blood Press Res (2020) 45(4):589–602. doi: 10.1159/000508372 32721950

[41] El-Lateef AEA El-Shemi AGA Alhammady MS Yuan R Zhang Y. LncRNA NEAT2 modulates pyroptosis of renal tubular cells induced by high glucose in diabetic nephropathy (DN) by *via* miR-206 regulation. Biochem Genet (2022) 60(5):1733–47. doi: 10.1007/s10528-021-10164-6 35084640

[42] Xu J Wang Q Song YF Xu XH Zhu H Chen PD. Long noncoding RNA X-inactive specific transcript regulates NLR family pyrin domain containing 3/caspase-1-mediated pyroptosis in diabetic nephropathy. World J Diabetes (2022) 13(4):358–75. doi: 10.4239/wjd.v13.i4.358 PMC9052004 35582664

[43] Zhu B Cheng X Jiang Y Cheng M Chen L Bao J. Silencing of KCNQ1OT1 decreases oxidative stress and pyroptosis of renal tubular epithelial cells. Diabetes Metab Syndr Obes (2020) 13:365–75. doi: 10.2147/DMSO.S225791 PMC7025682 32104033

[44] Xie C Wu W Tang A Luo N Tan Y. lncRNA GAS5/miR-452-5p reduces oxidative stress and pyroptosis of high-Glucose-Stimulated renal tubular cells. Diabetes Metab Syndr Obes (2019) 12:2609–17. doi: 10.2147/DMSO.S228654 PMC6910862 31849505

[45] Zhang C Gong Y Li N Liu X Zhang Y Ye F. Long noncoding RNA Kcnq1ot1 promotes sC5b-9-induced podocyte pyroptosis by inhibiting miR-486a-3p and upregulating NLRP3. Am J Physiol Cell Physiol (2021) 320(3):C355–C64. doi: 10.1152/ajpcell.00403.2020 33296289

[46] Wang J Zhao SM. LncRNA-antisense non-coding RNA in the INK4 locus promotes pyroptosis *via* miR-497/thioredoxin-interacting protein axis in diabetic nephropathy. Life Sci (2021) 264:118728. doi: 10.1016/j.lfs.2020.118728 33160992

[47] Yi H Peng R Zhang LY Sun Y Peng HM Liu HD. LincRNA-Gm4419 knockdown ameliorates NF-kappaB/NLRP3 inflammasome-mediated inflammation in diabetic nephropathy. Cell Death Dis (2017) 8(2):e2583. doi: 10.1038/cddis.2016.451 28151474 PMC5386454

[48] Wang Y Ding L Wang R Guo Y Yang Z Yu L. Circ_0004951 promotes pyroptosis of renal tubular cells *via* the NLRP3 inflammasome in diabetic kidney disease. Front Med (Lausanne) (2022) 9:828240. doi: 10.3389/fmed.2022.828240 35733856 PMC9207212

[49] Wu R Liu X Yin J Wu H Cai X Wang N. IL-6 receptor blockade ameliorates diabetic nephropathy *via* inhibiting inflammasome in mice. Metabolism (2018) 83:18–24. doi: 10.1016/j.metabol.2018.01.002 29336982

[50] Wang S Li Y Fan J Zhang X Luan J Bian Q. Interleukin-22 ameliorated renal injury and fibrosis in diabetic nephropathy through inhibition of NLRP3 inflammasome activation. Cell Death Dis (2017) 8(7):e2937. doi: 10.1038/cddis.2017.292 28726774 PMC5550847

[51] Zhang X Zhu Y Zhou Y Fei B. Interleukin 37 (IL-37) reduces high glucose-induced inflammation, oxidative stress, and apoptosis of podocytes by inhibiting the STAT3-cyclophilin a (CypA) signaling pathway. Med Sci Monit (2020) 26:e922979. doi: 10.12659/MSM.922979 32931486 PMC7518013

[52] Zhao J Chen J Li YY Xia LL Wu YG. Bruton’s tyrosine kinase regulates macrophage−induced inflammation in the diabetic kidney *via* NLRP3 inflammasome activation. Int J Mol Med (2021) 48(3):177. doi: 10.3892/ijmm.2021.5010 34278465 PMC8354311

[53] Purvis GSD Collino M Aranda-Tavio H Chiazza F O’Riordan CE Zeboudj L. Inhibition of bruton’s TK regulates macrophage NF-kappaB and NLRP3 inflammasome activation in metabolic inflammation. Br J Pharmacol (2020) 177(19):4416–32. doi: 10.1111/bph.15182 PMC7484557 32608058

[54] Wan J Jiang Z Liu D Pan S Zhou S Liu Z. Inhibition of the glycogen synthase kinase 3beta-hypoxia-inducible factor 1alpha pathway alleviates NLRP3-mediated pyroptosis induced by high glucose in renal tubular epithelial cells. Exp Physiol (2022) 107(12):1493–506. doi: 10.1113/EP090685 36056793

[55] Hou Q Kan S Wang Z Shi J Zeng C Yang D. Inhibition of HDAC6 with CAY10603 ameliorates diabetic kidney disease by suppressing NLRP3 inflammasome. Front Pharmacol (2022) 13:938391. doi: 10.3389/fphar.2022.938391 35910382 PMC9332914

[56] Shi Y Huang C Zhao Y Cao Q Yi H Chen X. RIPK3 blockade attenuates tubulointerstitial fibrosis in a mouse model of diabetic nephropathy. Sci Rep (2020) 10(1):10458. doi: 10.1038/s41598-020-67054-x 32591618 PMC7319952

[57] Qiao Y Tian X Men L Li S Chen Y Xue M. Spleen tyrosine kinase promotes NLR family pyrin domain containing 3 inflammasome−mediated IL−1β secretion *via* c−Jun n−terminal kinase activation and cell apoptosis during diabetic nephropathy. Mol Med Rep (2018) 18(2):1995–2008. doi: 10.3892/mmr.2018.9164 PMC6072182 29901140

[58] Collino M Benetti E Rogazzo M Mastrocola R Yaqoob MM Aragno M. Reversal of the deleterious effects of chronic dietary HFCS-55 intake by PPAR-delta agonism correlates with impaired NLRP3 inflammasome activation. Biochem Pharmacol (2013) 85(2):257–64. doi: 10.1016/j.bcp.2012.10.014 23103566

[59] Ying C Zhou Z Dai J Wang M Xiang J Sun D. Activation of the NLRP3 inflammasome by RAC1 mediates a new mechanism in diabetic nephropathy. Inflammation Res (2022) 71(2):191–204. doi: 10.1007/s00011-021-01532-4 35028708

[60] Wang B Dai Z Gao Q Liu Y Gu G Zheng H. Spop ameliorates diabetic nephropathy through restraining NLRP3 inflammasome. Biochem Biophys Res Commun (2022) 594:131–8. doi: 10.1016/j.bbrc.2021.12.068 35081502

[61] Lan J Xu B Shi X Pan Q Tao Q. WTAP-mediated N(6)-methyladenosine modification of NLRP3 mRNA in kidney injury of diabetic nephropathy. Cell Mol Biol Lett (2022) 27(1):51. doi: 10.1186/s11658-022-00350-8 35761192 PMC9235192

[62] Hinz M Scheidereit C. The IkappaB kinase complex in NF-kappaB regulation and beyond. EMBO Rep (2014) 15(1):46–61. doi: 10.1002/embr.201337983 24375677 PMC4303448

[63] Li F Song L Chen J Chen Y Li Y Huang M. Effect of genipin-1-β-d-gentiobioside on diabetic nephropathy in mice by activating AMP-activated protein kinase/silencing information regulator-related enzyme 1/nuclear factor-κB pathway. J Pharm Pharmacol (2021) 73(9):1201–11. doi: 10.1093/jpp/rgab041 33792721

[64] Pan M Zhang F Qu K Liu C Zhang J. TXNIP: a double-edged sword in disease and therapeutic outlook. Oxid Med Cell Longev (2022) 2022:7805115. doi: 10.1155/2022/7805115 35450411 PMC9017576

[65] Ludwig DL Kotanides H Le T Chavkin D Bohlen P Witte L. Cloning, genetic characterization, and chromosomal mapping of the mouse VDUP1 gene. Gene (2001) 269(1-2):103–12. doi: 10.1016/s0378-1119(01)00455-3 11376942

[66] Zhou R Tardivel A Thorens B Choi I Tschopp J. Thioredoxin-interacting protein links oxidative stress to inflammasome activation. Nat Immunol (2010) 11(2):136–40. doi: 10.1038/ni.1831 20023662

[67] Tonelli C Chio IIC Tuveson DA. Transcriptional regulation by Nrf2. Antioxid Redox Signal (2018) 29(17):1727–45. doi: 10.1089/ars.2017.7342 PMC6208165 28899199

[68] Silva-Islas CA Maldonado PD. Canonical and non-canonical mechanisms of Nrf2 activation. Pharmacol Res (2018) 134:92–9. doi: 10.1016/j.phrs.2018.06.013 29913224

[69] Hombach S Kretz M. Non-coding RNAs: classification, biology and functioning. Adv Exp Med Biol (2016) 937:3–17. doi: 10.1007/978-3-319-42059-2_1 27573892

[70] Kopp F Mendell JT. Functional classification and experimental dissection of long noncoding RNAs. Cell (2018) 172(3):393–407. doi: 10.1016/j.cell.2018.01.011 29373828 PMC5978744

[71] Wilusz JE Sunwoo H Spector DL. Long noncoding RNAs: functional surprises from the RNA world. Genes Dev (2009) 23(13):1494–504. doi: 10.1101/gad.1800909 PMC3152381 19571179

[72] Weber ANR Bittner Z Liu X Dang TM Radsak MP Brunner C. Bruton’s tyrosine kinase: an emerging key player in innate immunity. Front Immunol (2017) 8:1454. doi: 10.3389/fimmu.2017.01454 29167667 PMC5682317

[73] Woodgett JR Cohen P. Multisite phosphorylation of glycogen synthase. molecular basis for the substrate specificity of glycogen synthase kinase-3 and casein kinase-II (glycogen synthase kinase-5). Biochim Biophys Acta (1984) 788(3):339–47. doi: 10.1016/0167-4838(84)90047-5 6087911

[74] Mennerich D Dimova EY Kietzmann T. Direct phosphorylation events involved in HIF-alpha regulation: the role of GSK-3beta. Hypoxia (Auckl) (2014) 2:35–45. doi: 10.2147/HP.S60703 27774465 PMC5045055

[75] Miyake Y Keusch JJ Wang L Saito M Hess D Wang X. Structural insights into HDAC6 tubulin deacetylation and its selective inhibition. Nat Chem Biol (2016) 12(9):748–54. doi: 10.1038/nchembio.2140 27454931

[76] Wagner N Wagner KD. The role of PPARs in disease. Cells (2020) 9(11):2367. doi: 10.3390/cells9112367 33126411 PMC7692109

[77] Wada J Makino H. Innate immunity in diabetes and diabetic nephropathy. Nat Rev Nephrol (2016) 12(1):13–26. doi: 10.1038/nrneph.2015.175 26568190

[78] Kim D Li HY Lee JH Oh YS Jun HS. Lysophosphatidic acid increases mesangial cell proliferation in models of diabetic nephropathy *via* Rac1/MAPK/KLF5 signaling. Exp Mol Med (2019) 51(2):1–10. doi: 10.1038/s12276-019-0217-3 PMC6377648 30770784

[79] Zhuang M Calabrese MF Liu J Waddell MB Nourse A Hammel M. Structures of SPOP-substrate complexes: insights into molecular architectures of BTB-Cul3 ubiquitin ligases. Mol Cell (2009) 36(1):39–50. doi: 10.1016/j.molcel.2009.09.022 19818708 PMC2847577

[80] Guillamot M Ouazia D Dolgalev I Yeung ST Kourtis N Dai Y. The E3 ubiquitin ligase SPOP controls resolution of systemic inflammation by triggering MYD88 degradation. Nat Immunol (2019) 20(9):1196–207. doi: 10.1038/s41590-019-0454-6 PMC7376385 31406379

[81] Chen F Wei G Xu J Ma X Wang Q. Naringin ameliorates the high glucose-induced rat mesangial cell inflammatory reaction by modulating the NLRP3 inflammasome. BMC Complementary Altern Med (2018) 18(1):192. doi: 10.1186/s12906-018-2257-y PMC6014005 29929501

[82] Wang C Pan Y Zhang QY Wang FM Kong LD. Quercetin and allopurinol ameliorate kidney injury in STZ-treated rats with regulation of renal NLRP3 inflammasome activation and lipid accumulation. PloS One (2012) 7(6):e38285. doi: 10.1371/journal.pone.0038285 22701621 PMC3372527

[83] Ding T Wang S Zhang X Zai W Fan J Chen W. Kidney protection effects of dihydroquercetin on diabetic nephropathy through suppressing ROS and NLRP3 inflammasome. Phytomedicine (2018) 41:45–53. doi: 10.1016/j.phymed.2018.01.026 29519318

[84] Dong W Jia C Li J Zhou Y Luo Y Liu J. Fisetin attenuates diabetic nephropathy-induced podocyte injury by inhibiting NLRP3 inflammasome. Front Pharmacol (2022) 13:783706. doi: 10.3389/fphar.2022.783706 35126159 PMC8816314

[85] Ge C Xu M Qin Y Gu T Lou D Li Q. Fisetin supplementation prevents high fat diet-induced diabetic nephropathy by repressing insulin resistance and RIP3-regulated inflammation. Food Funct (2019) 10(5):2970–85. doi: 10.1039/c8fo01653d 31074472

[86] Zhu X Shi J Li H. Liquiritigenin attenuates high glucose-induced mesangial matrix accumulation, oxidative stress, and inflammation by suppression of the NF-κB and NLRP3 inflammasome pathways. Biomed Pharmacother (2018) 106:976–82. doi: 10.1016/j.biopha.2018.07.045 30119269

[87] Alzahrani S Zaitone SA Said E El-Sherbiny M Ajwah S Alsharif SY. Protective effect of isoliquiritigenin on experimental diabetic nephropathy in rats: impact on sirt-1/NFkappaB balance and NLRP3 expression. Int Immunopharmacol (2020) 87:106813. doi: 10.1016/j.intimp.2020.106813 32707499

[88] Yosri H El-Kashef DH El-Sherbiny M Said E Salem HA. Calycosin modulates NLRP3 and TXNIP-mediated pyroptotic signaling and attenuates diabetic nephropathy progression in diabetic rats; an insight. BioMed Pharmacother (2022) 155:113758. doi: 10.1016/j.biopha.2022.113758 36271546

[89] Yu Q Zhang M Qian L Wen D Wu G. Luteolin attenuates high glucose-induced podocyte injury *via* suppressing NLRP3 inflammasome pathway. Life Sci (2019) 225:1–7. doi: 10.1016/j.lfs.2019.03.073 30935950

[90] Ren C Bao X Lu X Du W Wang X Wei J. Complanatoside a targeting NOX4 blocks renal fibrosis in diabetic mice by suppressing NLRP3 inflammasome activation and autophagy. Phytomedicine (2022) 104:154310. doi: 10.1016/j.phymed.2022.154310 35843189

[91] Tang L Fang C Wang H Tang S. The protective effect of kaempferol on renal function and tissue of diabetic nephropathy rats induced by high glucose. Immunol J (2018) 34(12):1041–6. doi: 10.13431/j.cnki.immunol.j.20180162

[92] Gao Y Tan H Chang X Liu N Zheng P Yuan L. Protective effect of scarithamine on diabetic nephropathy. Chin J Gerontology (2018) 38(6):1433–5.

[93] Wang T Gao Y Yue R Wang X Shi Y Xu J. Ginsenoside Rg1 alleviates podocyte injury induced by hyperlipidemia *via* targeting the mTOR/NF-kappaB/NLRP3 axis. Evid Based Complement Alternat Med (2020) 2020:2735714. doi: 10.1155/2020/2735714 33133213 PMC7568787

[94] Zhu Y Zhu C Yang H Deng J Fan D. Protective effect of ginsenoside Rg5 against kidney injury *via* inhibition of NLRP3 inflammasome activation and the MAPK signaling pathway in high-fat diet/streptozotocin-induced diabetic mice. Pharmacol Res (2020) 155:104746. doi: 10.1016/j.phrs.2020.104746 32156650

[95] Song W Wei L Du Y Wang Y Jiang S. Protective effect of ginsenoside metabolite compound K against diabetic nephropathy by inhibiting NLRP3 inflammasome activation and NF-κB/p38 signaling pathway in high-fat diet/streptozotocin-induced diabetic mice. Int Immunopharmacol (2018) 63:227–38. doi: 10.1016/j.intimp.2018.07.027 30107367

[96] Tang ZZ Zhang YM Zheng T Huang TT Ma TF Liu YW. Sarsasapogenin alleviates diabetic nephropathy through suppression of chronic inflammation by down-regulating PAR-1: *In vivo* and *in vitro* study. Phytomedicine (2020) 78:153314. doi: 10.1016/j.phymed.2020.153314 32882582

[97] Liu YW Hao YC Chen YJ Yin SY Zhang MY Kong L. Protective effects of sarsasapogenin against early stage of diabetic nephropathy in rats. Phytother Res (2018) 32(8):1574–82. doi: 10.1002/ptr.6088 29682805

[98] Feng H Zhu X Tang Y Fu S Kong B Liu X. Astragaloside IV ameliorates diabetic nephropathy in db/db mice by inhibiting NLRP3 inflammasome−mediated inflammation. Int J Mol Med (2021) 48(2):164. doi: 10.3892/ijmm.2021.4996 34278447 PMC8262660

[99] Zhao J Zhang L Kang H. Inhibitory effect and mechanism of astragaloside IV on NLRP3 inflammasome activation pathway in mesangial cells of diabetic nephropathy based on the autophagy pathway. Guiding J Traditional Chin Med Pharmacol (2021) 27(9):41–6. doi: 10.13862/j.cnki.cn43-1446/r.2021.09.011

[100] Wang S Zhao X Yang S Chen B Shi J. Salidroside alleviates high glucose-induced oxidative stress and extracellular matrix accumulation in rat glomerular mesangial cells by the TXNIP-NLRP3 inflammasome pathway. Chem Biol Interact (2017) 278:48–53. doi: 10.1016/j.cbi.2017.10.012 29031534

[101] Zheng L Xue R Lv T Ge S Gui D Wang N. Study on renal protective effect of notoginsenoside fc on db/db mice. Chin Arch Tranditional Chin Med (2017) 35(3):609–22. doi: 10.13193/j.issn.1673-7717.2017.03.026

[102] Li J Wang B Zhou G Yan X Zhang Y. Tetrahydroxy stilbene glucoside alleviates high glucose-induced MPC5 podocytes injury through suppression of NLRP3 inflammasome. Am J Med Sci (2018) 355(6):588–96. doi: 10.1016/j.amjms.2018.03.005 29891042

[103] Huang L Shao M Zhu Y. Gastrodin inhibits high glucose-induced inflammation, oxidative stress and apoptosis in podocytes by activating the AMPK/Nrf2 signaling pathway. Exp Ther Med (2022) 23(2):168. doi: 10.3892/etm.2021.11091 35069849 PMC8753962

[104] Yang R Chen J Jia Q Yang X Mehmood S. Epigallocatechin-3-gallate ameliorates renal endoplasmic reticulum stress-mediated inflammation in type 2 diabetic rats. Exp Biol Med (Maywood) (2022) 247(16):1410–9. doi: 10.1177/15353702221106479 PMC9493765 35775606

[105] Xiao YD Huang YY Wang HX Wu Y Leng Y Liu M. Thioredoxin-interacting protein mediates NLRP3 inflammasome activation involved in the susceptibility to ischemic acute kidney injury in diabetes. Oxid Med Cell Longev (2016) 2016:2386068. doi: 10.1155/2016/2386068 27867451 PMC5102753

[106] Yao C. Piceatannol attenuates high glucose-induced podocyte injury via Nrf2-mediated inhibition of NLRP3 inflammasome. Zhengzhou, Henan, China: Zhengzhou University (2018).

[107] Lu M Yin N Liu W Cui X Chen S Wang E. Curcumin ameliorates diabetic nephropathy by suppressing NLRP3 inflammasome signaling. BioMed Res Int (2017) 2017:1516985. doi: 10.1155/2017/1516985 28194406 PMC5282455

[108] An X Zhang Y Cao Y Chen J Qin H Yang L. Punicalagin protects diabetic nephropathy by inhibiting pyroptosis based on TXNIP/NLRP3 pathway. Nutrients (2020) 12(5):1516. doi: 10.3390/nu12051516 32456088 PMC7284711

[109] Zheng GH Shan Q Mu JJ Wang YJ Zhang ZF Fan SH. Purple sweet potato color attenuates kidney damage by blocking VEGFR2/ROS/NLRP3 signaling in high-fat diet-treated mice. Oxid Med Cell Longev (2019) 2019:5189819. doi: 10.1155/2019/5189819 30805082 PMC6360596

[110] Qiu T Wang D. Effects of grape seed proanthocyanidins on renal ischemia-reperfusion injury in diabetic rats. Chin J Clin Pharmacol (2020) 36(14):2036–8. doi: 10.13699/j.cnki.1001-6821.2020.14.034

[111] Zhao Q Bi Y Guo J Liu YX Zhong J Pan LR. Pristimerin protects against inflammation and metabolic disorder in mice through inhibition of NLRP3 inflammasome activation. Acta Pharmacol Sin (2021) 42(6):975–86. doi: 10.1038/s41401-020-00527-x PMC8149413 32989235

[112] Li F Chen Y Li Y Huang M Zhao W. Geniposide alleviates diabetic nephropathy of mice through AMPK/SIRT1/NF-kappaB pathway. Eur J Pharmacol (2020) 886:173449. doi: 10.1016/j.ejphar.2020.173449 32758570

[113] Duan J He L Deng W Lu M Zhai Y Pei F. Natural swietenine attenuates diabetic nephropathy by regulating the NF-kappaB/NLRP3/Caspase-1 signaling pathways: *In vivo* and *in vitro* study. Environ Toxicol (2022) 37(12):2977–89. doi: 10.1002/tox.23653 36066211

[114] Sun Z Ma Y Chen F Wang S Chen B Shi J. Artesunate ameliorates high glucose-induced rat glomerular mesangial cell injury by suppressing the TLR4/NF-κB/NLRP3 inflammasome pathway. Chemico-Biological Interactions (2018) 293:11–9. doi: 10.1016/j.cbi.2018.07.011 30031708

[115] Chen J Yang Y Lv Z Shu A Du Q Wang W. Study on the inhibitive effect of catalpol on diabetic nephropathy. Life Sci (2020) 257:118120. doi: 10.1016/j.lfs.2020.118120 32693244

[116] Liu W Liang L Zhang Q Li Y Yan S Tang T. Effects of andrographolide on renal tubulointersticial injury and fibrosis. Evidence its Mech action Phytomedicine (2021) 91:153650. doi: 10.1016/j.phymed.2021.153650 34332282

[117] Wu W Liu B Wan Y Sun W Liu Y Wang W. Triptolide inhibits NLRP3 inflammasome activation and ameliorates podocyte epithelial-mesenchymal transition induced by high glucose. J Chin Materia Med (2019) 44(24):5457–64. doi: 10.19540/j.cnki.cjcmm.20191114.401 32237395

[118] Ma Z Zhu L Wang S Guo X Sun B Wang Q. Berberine protects diabetic nephropathy by suppressing epithelial-to-mesenchymal transition involving the inactivation of the NLRP3 inflammasome. Ren Fail (2022) 44(1):923–32. doi: 10.1080/0886022X.2022.2079525 PMC9154812 35618411

[119] Samra YA Said HS Elsherbiny NM Liou GI El-Shishtawy MM Eissa LA. Cepharanthine and piperine ameliorate diabetic nephropathy in rats: role of NF-kappaB and NLRP3 inflammasome. Life Sci (2016) 157:187–99. doi: 10.1016/j.lfs.2016.06.002 27266851

[120] Zhang Q Hu Y Hu JE Zhang M. Solasonine alleviates high glucose-induced podocyte injury through increasing Nrf2-medicated inhibition of NLRP3 activation. Drug Dev Res (2022) 83(7):1697–706. doi: 10.1002/ddr.21988 36048966

[121] Hu X. Effects and mechanisms of rutaecarpine on podocyte injury on diabetic kidney disease. Hefei, Anhui, China: Anhui Medical University (2022).

[122] Wang X Li Q Sui B Xu M Pu Z Qiu T. Schisandrin a from schisandra chinensis attenuates ferroptosis and NLRP3 inflammasome-mediated pyroptosis in diabetic nephropathy through mitochondrial damage by AdipoR1 ubiquitination. Oxid Med Cell Longev (2022) 2022:5411462. doi: 10.1155/2022/5411462 35996380 PMC9391610

[123] Ma R He Y Fang Q Xie G Qi M. Ferulic acid ameliorates renal injury *via* improving autophagy to inhibit inflammation in diabetic nephropathy mice. BioMed Pharmacother (2022) 153:113424. doi: 10.1016/j.biopha.2022.113424 36076545

[124] Yoon JJ Lee HK Kim HY Han BH Lee HS Lee YJ. Sauchinone protects renal mesangial cell dysfunction against angiotensin II by improving renal fibrosis and inflammation. Int J Mol Sci (2020) 21(19):7003. doi: 10.3390/ijms21197003 32977573 PMC7583825

[125] Zhang L Jing M Liu Q. Crocin alleviates the inflammation and oxidative stress responses associated with diabetic nephropathy in rats *via* NLRP3 inflammasomes. Life Sci (2021) 278:119542. doi: 10.1016/j.lfs.2021.119542 33915128

[126] Qu X Zhai B Liu Y Chen Y Xie Z Wang Q. Pyrroloquinoline quinone ameliorates renal fibrosis in diabetic nephropathy by inhibiting the pyroptosis pathway in C57BL/6 mice and human kidney 2 cells. BioMed Pharmacother (2022) 150:112998. doi: 10.1016/j.biopha.2022.112998 35489281

[127] Xin R Sun X Wang Z Yuan W Jiang W Wang L. Apocynin inhibited NLRP3/XIAP signalling to alleviate renal fibrotic injury in rat diabetic nephropathy. Biomed Pharmacother (2018) 106:1325–31. doi: 10.1016/j.biopha.2018.07.036 30119203

[128] Shen J Zhu H Hu F Yan Z Li Y Zhong S. Effect of diallyl trisulfide on pyroptosis in renal tissue of diabetic nephropathy rats. J Guangzhou Univ Traditional Chin Med (2022) 39(7):1643–50. doi: 10.13359/j.cnki.gzxbtcm.2022.07.031

[129] Dias MC Pinto D Silva AMS. Plant flavonoids: chemical characteristics and biological activity. Molecules (2021) 26(17):5377. doi: 10.3390/molecules26175377 34500810 PMC8434187

[130] Sadowska-Bartosz I Galiniak S Bartosz G. Polyphenols protect against protein glycoxidation. Free Radic Biol Med (2014) 75 Suppl 1:S47. doi: 10.1016/j.freeradbiomed.2014.10.810 26461390

[131] Dhanya R Arun KB Nisha VM Syama HP Nisha P Santhosh Kumar TR. Preconditioning L6 muscle cells with naringin ameliorates oxidative stress and increases glucose uptake. PloS One (2015) 10(7):e0132429. doi: 10.1371/journal.pone.0132429 26147673 PMC4492986

[132] Kandhare AD Ghosh P Bodhankar SL. Naringin, a flavanone glycoside, promotes angiogenesis and inhibits endothelial apoptosis through modulation of inflammatory and growth factor expression in diabetic foot ulcer in rats. Chem Biol Interact (2014) 219:101–12. doi: 10.1016/j.cbi.2014.05.012 24880026

[133] Hosseini A Razavi BM Banach M Hosseinzadeh H. Quercetin and metabolic syndrome: a review. Phytother Res (2021) 35(10):5352–64. doi: 10.1002/ptr.7144 34101925

[134] Yang P Xu F Li HF Wang Y Li FC Shang MY. Detection of 191 taxifolin metabolites and their distribution in rats using HPLC-ESI-IT-TOF-MS(n). Molecules (2016) 21(9):1209. doi: 10.3390/molecules21091209 27649117 PMC6273498

[135] Ebrahimpour S Zakeri M Esmaeili A. Crosstalk between obesity, diabetes, and alzheimer’s disease: introducing quercetin as an effective triple herbal medicine. Ageing Res Rev (2020) 62:101095. doi: 10.1016/j.arr.2020.101095 32535272

[136] Khursheed R Singh SK Wadhwa S Gulati M Awasthi A. Enhancing the potential preclinical and clinical benefits of quercetin through novel drug delivery systems. Drug Discov Today (2020) 25(1):209–22. doi: 10.1016/j.drudis.2019.11.001 31707120

[137] Patel RV Mistry BM Shinde SK Syed R Singh V Shin HS. Therapeutic potential of quercetin as a cardiovascular agent. Eur J Med Chem (2018) 155:889–904. doi: 10.1016/j.ejmech.2018.06.053 29966915

[138] Yang D Wang T Long M Li P. Quercetin: its main pharmacological activity and potential application in clinical medicine. Oxid Med Cell Longev (2020) 2020:8825387. doi: 10.1155/2020/8825387 33488935 PMC7790550

[139] Weidmann AE. Dihydroquercetin: more than just an impurity? Eur J Pharmacol (2012) 684(1-3):19–26. doi: 10.1016/j.ejphar.2012.03.035 22513183

[140] Li Z Deng H Guo X Yan S Lu C Zhao Z. Effective dose/duration of natural flavonoid quercetin for treatment of diabetic nephropathy: a systematic review and meta-analysis of rodent data. Phytomedicine (2022) 105:154348. doi: 10.1016/j.phymed.2022.154348 35908521

[141] Rengarajan T Yaacob NS. The flavonoid fisetin as an anticancer agent targeting the growth signaling pathways. Eur J Pharmacol (2016) 789:8–16. doi: 10.1016/j.ejphar.2016.07.001 27377217

[142] Dong B Liu C Xue R Wang Y Sun Y Liang Z. Fisetin inhibits cardiac hypertrophy by suppressing oxidative stress. J Nutr Biochem (2018) 62:221–9. doi: 10.1016/j.jnutbio.2018.08.010 30312797

[143] Zhang J Zhao L Hu C Wang T Lu J Wu C. Fisetin prevents acetaminophen-induced liver injury by promoting autophagy. Front Pharmacol (2020) 11:162. doi: 10.3389/fphar.2020.00162 32184730 PMC7058798

[144] Asl MN Hosseinzadeh H. Review of pharmacological effects of glycyrrhiza sp. and its bioactive compounds. Phytother Res (2008) 22(6):709–24. doi: 10.1002/ptr.2362 PMC7167813 18446848

[145] Zhou T Deng X Qiu J. Antimicrobial activity of licochalcone e against staphylococcus aureus and its impact on the production of staphylococcal alpha-toxin. J Microbiol Biotechnol (2012) 22(6):800–5. doi: 10.4014/jmb.1112.12020 22573157

[146] Yu JY Ha JY Kim KM Jung YS Jung JC Oh S. Anti-inflammatory activities of licorice extract and its active compounds, glycyrrhizic acid, liquiritin and liquiritigenin, in BV2 cells and mice liver. Molecules (2015) 20(7):13041–54. doi: 10.3390/molecules200713041 PMC6332102 26205049

[147] Park SM Lee JR Ku SK Cho IJ Byun SH Kim SC. Isoliquiritigenin in licorice functions as a hepatic protectant by induction of antioxidant genes through extracellular signal-regulated kinase-mediated NF-E2-related factor-2 signaling pathway. Eur J Nutr (2016) 55(8):2431–44. doi: 10.1007/s00394-015-1051-6 26593436

[148] Chen C Huang S Chen CL Su SB Fang DD. Isoliquiritigenin inhibits ovarian cancer metastasis by reversing epithelial-to-Mesenchymal transition. Molecules (2019) 24(20):3725. doi: 10.3390/molecules24203725 31623144 PMC6833095

[149] He C Wang Z Shi J. Pharmacological effects of icariin. Adv Pharmacol (2020) 87:179–203. doi: 10.1016/bs.apha.2019.10.004 32089233

[150] Ren M Wang X Du G Tian J Liu Y. Calycosin−7−O−beta−D−glucoside attenuates ischemia−reperfusion injury *in vivo via* activation of the PI3K/Akt pathway. Mol Med Rep (2016) 13(1):633–40. doi: 10.3892/mmr.2015.4611 PMC4686071 26648122

[151] Elsherbiny NM Said E Atef H Zaitone SA. Renoprotective effect of calycosin in high fat diet-fed/STZ injected rats: effect on IL-33/ST2 signaling, oxidative stress and fibrosis suppression. Chem Biol Interact (2020) 315:108897. doi: 10.1016/j.cbi.2019.108897 31726037

[152] Liu Y Huang J Zheng X Yang X Ding Y Fang T. Luteolin, a natural flavonoid, inhibits methylglyoxal induced apoptosis *via* the mTOR/4E-BP1 signaling pathway. Sci Rep (2017) 7(1):7877. doi: 10.1038/s41598-017-08204-6 28801605 PMC5554232

[153] Choi BM Lim DW Lee JA Gao SS Kwon DY Kim BR. Luteolin suppresses cisplatin-induced apoptosis in auditory cells: possible mediation through induction of heme oxygenase-1 expression. J Med Food (2008) 11(2):230–6. doi: 10.1089/jmf.2007.591 18598163

[154] Aziz N Kim MY Cho JY. Anti-inflammatory effects of luteolin: a review of *in vitro*, *in vivo*, and in silico studies. J Ethnopharmacol (2018) 225:342–58. doi: 10.1016/j.jep.2018.05.019 29801717

[155] Ng YF Tang PC Sham TT Lam WS Mok DK Chan SW. Semen astragali complanati: an ethnopharmacological, phytochemical and pharmacological review. J Ethnopharmacol (2014) 155(1):39–53. doi: 10.1016/j.jep.2014.06.013 24933224

[156] Imran M Salehi B Sharifi-Rad J Aslam Gondal T Saeed F Imran A. Kaempferol: a key emphasis to its anticancer potential. Molecules (2019) 24(12):2277. doi: 10.3390/molecules24122277 31248102 PMC6631472

[157] Devi KP Malar DS Nabavi SF Sureda A Xiao J Nabavi SM. Kaempferol and inflammation: from chemistry to medicine. Pharmacol Res (2015) 99:1–10. doi: 10.1016/j.phrs.2015.05.002 25982933

[158] Guo H Zhu L Tang P Chen D Li Y Li J. Carthamin yellow improves cerebral ischemia−reperfusion injury by attenuating inflammation and ferroptosis in rats. Int J Mol Med (2021) 47(4):52. doi: 10.3892/ijmm.2021.4885 33576458 PMC7895518

[159] Singh D Chaudhuri PK. Structural characteristics, bioavailability and cardioprotective potential of saponins. Integr Med Res (2018) 7(1):33–43. doi: 10.1016/j.imr.2018.01.003 29629289 PMC5884006

[160] Yang WZ Hu Y Wu WY Ye M Guo DA. Saponins in the genus panax l. (Araliaceae): a systematic review of their chemical diversity. Phytochemistry (2014) 106:7–24. doi: 10.1016/j.phytochem.2014.07.012 25108743

[161] Coon JT Ernst E. Panax ginseng: a systematic review of adverse effects and drug interactions. Drug Saf (2002) 25(5):323–44. doi: 10.2165/00002018-200225050-00003 12020172

[162] Nakata H Kikuchi Y Tode T Hirata J Kita T Ishii K. Inhibitory effects of ginsenoside Rh2 on tumor growth in nude mice bearing human ovarian cancer cells. Jpn J Cancer Res (1998) 89(7):733–40. doi: 10.1111/j.1349-7006.1998.tb03278.x PMC5921889 9738980

[163] Zhou W Chai H Lin PH Lumsden AB Yao Q Chen CJ. Molecular mechanisms and clinical applications of ginseng root for cardiovascular disease. Med Sci Monit (2004) 10(8):RA187–92. 15278009

[164] Xie JT Mehendale SR Li X Quigg R Wang X Wang CZ. Anti-diabetic effect of ginsenoside re in ob/ob mice. Biochim Biophys Acta (2005) 1740(3):319–25. doi: 10.1016/j.bbadis.2004.10.010 15949698

[165] Cho JH Chun HY Lee JS Lee JH Cheong KJ Jung YS. Prevention effect of rare ginsenosides against stress-hormone induced MTOC amplification. Oncotarget (2016) 7(23):35144–58. doi: 10.18632/oncotarget.9059 PMC5085216 27147573

[166] Liang LD He T Du TW Fan YG Chen DS Wang Y. Ginsenoside−Rg5 induces apoptosis and DNA damage in human cervical cancer cells. Mol Med Rep (2015) 11(2):940–6. doi: 10.3892/mmr.2014.2821 PMC4262516 25355274

[167] Yang XD Yang YY Ouyang DS Yang GP. A review of biotransformation and pharmacology of ginsenoside compound K. Fitoterapia (2015) 100:208–20. doi: 10.1016/j.fitote.2014.11.019 25449425

[168] Kang LP Zhang J Cong Y Li B Xiong CQ Zhao Y. Steroidal glycosides from the rhizomes of anemarrhena asphodeloides and their antiplatelet aggregation activity. Planta Med (2012) 78(6):611–6. doi: 10.1055/s-0031-1298223 22307934

[169] Kim JY Shin JS Ryu JH Kim SY Cho YW Choi JH. Anti-inflammatory effect of anemarsaponin b isolated from the rhizomes of anemarrhena asphodeloides in LPS-induced RAW 264.7 macrophages is mediated by negative regulation of the nuclear factor-kappaB and p38 pathways. Food Chem Toxicol (2009) 47(7):1610–7. doi: 10.1016/j.fct.2009.04.009 19375480

[170] Lim SM Jeong JJ Kang GD Kim KA Choi HS Kim DH. Timosaponin AIII and its metabolite sarsasapogenin ameliorate colitis in mice by inhibiting NF-kappaB and MAPK activation and restoring Th17/Treg cell balance. Int Immunopharmacol (2015) 25(2):493–503. doi: 10.1016/j.intimp.2015.02.016 25698557

[171] Wang Z Cai J Fu Q Cheng L Wu L Zhang W. Anti-inflammatory activities of compounds isolated from the rhizome of anemarrhena asphodeloides. Molecules (2018) 23(10):2631. doi: 10.3390/molecules23102631 30322157 PMC6222787

[172] Zhang Z Wang J Zhu Y Zhang H Wang H. Astragaloside IV alleviates myocardial damage induced by type 2 diabetes *via* improving energy metabolism. Mol Med Rep (2019) 20(5):4612–22. doi: 10.3892/mmr.2019.10716 PMC6797977 31702040

[173] Li L Hou X Xu R Liu C Tu M. Research review on the pharmacological effects of astragaloside IV. Fundam Clin Pharmacol (2017) 31(1):17–36. doi: 10.1111/fcp.12232 27567103

[174] Ni GL Cui R Shao AM Wu ZM. Salidroside ameliorates diabetic neuropathic pain in rats by inhibiting neuroinflammation. J Mol Neurosci (2017) 63(1):9–16. doi: 10.1007/s12031-017-0951-8 28741143

[175] Zhuang X Maimaitijiang A Li Y Shi H Jiang X. Salidroside inhibits high-glucose induced proliferation of vascular smooth muscle cells *via* inhibiting mitochondrial fission and oxidative stress. Exp Ther Med (2017) 14(1):515–24. doi: 10.3892/etm.2017.4541 PMC5488502 28672961

[176] Wu YL Lian LH Jiang YZ Nan JX. Hepatoprotective effects of salidroside on fulminant hepatic failure induced by d-galactosamine and lipopolysaccharide in mice. J Pharm Pharmacol (2009) 61(10):1375–82. doi: 10.1211/jpp/61.10.0015 19814871

[177] Yin D Yao W Chen S Hu R Gao X. Salidroside, the main active compound of rhodiola plants, inhibits high glucose-induced mesangial cell proliferation. Planta Med (2009) 75(11):1191–5. doi: 10.1055/s-0029-1185717 19444770

[178] He C Li J Xu N Wang R Li Z Yang L. Pharmacokinetics, bioavailability, and metabolism of notoginsenoside fc in rats by liquid chromatography/electrospray ionization tandem mass spectrometry. J Pharm BioMed Anal (2015) 109:150–7. doi: 10.1016/j.jpba.2015.02.038 25770412

[179] Simon J Casado-Andres M Goikoetxea-Usandizaga N Serrano-Macia M Martinez-Chantar ML. Nutraceutical properties of polyphenols against liver diseases. Nutrients (2020) 12(11):3517. doi: 10.3390/nu12113517 33203174 PMC7697723

[180] Li F Zhang T He Y Gu W Yang X Zhao R. Inflammation inhibition and gut microbiota regulation by TSG to combat atherosclerosis in ApoE(-/-) mice. J Ethnopharmacol (2020) 247:112232. doi: 10.1016/j.jep.2019.112232 31606534

[181] Xu CB Guo QL Wang YN Lin S Zhu CG Shi JG. Gastrodin derivatives from gastrodia elata. Nat Prod Bioprospect (2019) 9(6):393–404. doi: 10.1007/s13659-019-00224-1 31734866 PMC6872707

[182] Liu Y Gao J Peng M Meng H Ma H Cai P. A review on central nervous system effects of gastrodin. Front Pharmacol (2018) 9:24. doi: 10.3389/fphar.2018.00024 29456504 PMC5801292

[183] Bertelli AA Das DK. Grapes, wines, resveratrol, and heart health. J Cardiovasc Pharmacol (2009) 54(6):468–76. doi: 10.1097/FJC.0b013e3181bfaff3 19770673

[184] Kitada M Koya D. Renal protective effects of resveratrol. Oxid Med Cell Longev (2013) 2013:568093. doi: 10.1155/2013/568093 24379901 PMC3863562

[185] Kitada M Kume S Imaizumi N Koya D. Resveratrol improves oxidative stress and protects against diabetic nephropathy through normalization of Mn-SOD dysfunction in AMPK/SIRT1-independent pathway. Diabetes (2011) 60(2):634–43. doi: 10.2337/db10-0386 PMC3028365 21270273

[186] Piotrowska H Kucinska M Murias M. Biological activity of piceatannol: leaving the shadow of resveratrol. Mutat Res (2012) 750(1):60–82. doi: 10.1016/j.mrrev.2011.11.001 22108298

[187] Banik K Ranaware AM Harsha C Nitesh T Girisa S Deshpande V. Piceatannol: a natural stilbene for the prevention and treatment of cancer. Pharmacol Res (2020) 153:104635. doi: 10.1016/j.phrs.2020.104635 31926274

[188] Wang S Wang G Wu W Xu Z Yang J Cao M. Autophagy activation by dietary piceatannol enhances the efficacy of immunogenic chemotherapy. Front Immunol (2022) 13:968686. doi: 10.3389/fimmu.2022.968686 35979349 PMC9376326

[189] Kershaw JC Elzey BD Guo XX Kim KH. Piceatannol, a dietary polyphenol, alleviates adipose tissue loss in pre-clinical model of cancer-associated cachexia *via* lipolysis inhibition. Nutrients (2022) 14(11):2306. doi: 10.3390/nu14112306 35684106 PMC9183120

[190] Tang YL Chan SW. A review of the pharmacological effects of piceatannol on cardiovascular diseases. Phytother Res (2014) 28(11):1581–8. doi: 10.1002/ptr.5185 24919577

[191] Ghosh S Banerjee S Sil PC. The beneficial role of curcumin on inflammation, diabetes and neurodegenerative disease: a recent update. Food Chem Toxicol (2015) 83:111–24. doi: 10.1016/j.fct.2015.05.022 26066364

[192] Suresh Babu P Srinivasan K. Amelioration of renal lesions associated with diabetes by dietary curcumin in streptozotocin diabetic rats. Mol Cell Biochem (1998) 181(1-2):87–96. doi: 10.1023/a:1006821828706 9562245

[193] Sharma S Kulkarni SK Chopra K. Curcumin, the active principle of turmeric (Curcuma longa), ameliorates diabetic nephropathy in rats. Clin Exp Pharmacol Physiol (2006) 33(10):940–5. doi: 10.1111/j.1440-1681.2006.04468.x 17002671

[194] Yoshida T Amakura Y Yoshimura M. Structural features and biological properties of ellagitannins in some plant families of the order myrtales. Int J Mol Sci (2010) 11(1):79–106. doi: 10.3390/ijms11010079 20162003 PMC2820991

[195] Berdowska I Matusiewicz M Fecka I. Punicalagin in cancer prevention-via signaling pathways targeting. Nutrients (2021) 13(8):2733. doi: 10.3390/nu13082733 34444893 PMC8400644

[196] Cao Y Chen J Ren G Zhang Y Tan X Yang L. Punicalagin prevents inflammation in LPS-induced RAW264.7 macrophages by inhibiting FoxO3a/Autophagy signaling pathway. Nutrients (2019) 11(11):2794. doi: 10.3390/nu11112794 31731808 PMC6893462

[197] Xu J Cao K Liu X Zhao L Feng Z Liu J. Punicalagin regulates signaling pathways in inflammation-associated chronic diseases. Antioxidants (Basel) (2021) 11(1):29. doi: 10.3390/antiox11010029 35052533 PMC8773334

[198] Shan Q Zheng Y Lu J Zhang Z Wu D Fan S. Purple sweet potato color ameliorates kidney damage *via* inhibiting oxidative stress mediated NLRP3 inflammasome activation in high fat diet mice. Food Chem Toxicol (2014) 69:339–46. doi: 10.1016/j.fct.2014.04.033 24795233

[199] Zhang ZF Fan SH Zheng YL Lu J Wu DM Shan Q. Purple sweet potato color attenuates oxidative stress and inflammatory response induced by d-galactose in mouse liver. Food Chem Toxicol (2009) 47(2):496–501. doi: 10.1016/j.fct.2008.12.005 19114082

[200] Sun C Fan S Wang X Lu J Zhang Z Wu D. Purple sweet potato color inhibits endothelial premature senescence by blocking the NLRP3 inflammasome. J Nutr Biochem (2015) 26(10):1029–40. doi: 10.1016/j.jnutbio.2015.04.012 26164602

[201] Bagchi D Bagchi M Stohs SJ Das DK Ray SD Kuszynski CA. Free radicals and grape seed proanthocyanidin extract: importance in human health and disease prevention. Toxicology (2000) 148(2-3):187–97. doi: 10.1016/s0300-483x(00)00210-9 10962138

[202] Rozanska D Regulska-Ilow B. The significance of anthocyanins in the prevention and treatment of type 2 diabetes. Adv Clin Exp Med (2018) 27(1):135–42. doi: 10.17219/acem/64983 29521054

[203] Li D Zhang Y Liu Y Sun R Xia M. Purified anthocyanin supplementation reduces dyslipidemia, enhances antioxidant capacity, and prevents insulin resistance in diabetic patients. J Nutr (2015) 145(4):742–8. doi: 10.3945/jn.114.205674 25833778

[204] Couillaud J Leydet L Duquesne K Iacazio G. The terpene mini-path, a new promising alternative for terpenoids bio-production. Genes (Basel) (2021) 12(12):1974. doi: 10.3390/genes12121974 34946923 PMC8701039

[205] Petronelli A Pannitteri G Testa U. Triterpenoids as new promising anticancer drugs. Anticancer Drugs (2009) 20(10):880–92. doi: 10.1097/CAD.0b013e328330fd90 19745720

[206] Yousef BA Hassan HM Zhang LY Jiang ZZ. Anticancer potential and molecular targets of pristimerin: a mini- review. Curr Cancer Drug Targets (2017) 17(2):100–8. doi: 10.2174/1568009616666160112105824 26758533

[207] Zhao Q Liu Y Zhong J Bi Y Liu Y Ren Z. Pristimerin induces apoptosis and autophagy *via* activation of ROS/ASK1/JNK pathway in human breast cancer *in vitro* and in vivo. Cell Death Discovery (2019) 5:125. doi: 10.1038/s41420-019-0208-0 31396402 PMC6680048

[208] Zhao Q Bi Y Zhong J Ren Z Liu Y Jia J. Pristimerin suppresses colorectal cancer through inhibiting inflammatory responses and wnt/beta-catenin signaling. Toxicol Appl Pharmacol (2020) 386:114813. doi: 10.1016/j.taap.2019.114813 31715269

[209] Hui B Zhang L Zhou Q Hui L. Pristimerin inhibits LPS-triggered neurotoxicity in BV-2 microglia cells through modulating IRAK1/TRAF6/TAK1-mediated NF-kappaB and AP-1 signaling pathways in vitro. Neurotox Res (2018) 33(2):268–83. doi: 10.1007/s12640-017-9837-3 29119451

[210] Wang J De-Qiong X Hong DQ Zhang QQ Zhang J. Attenuation of myocardial ischemia reperfusion injury by geniposide preconditioning in diabetic rats. Curr Res Transl Med (2019) 67(2):35–40. doi: 10.1016/j.retram.2019.03.002 30902610

[211] Liu JH Yin F Guo LX Deng XH Hu YH. Neuroprotection of geniposide against hydrogen peroxide induced PC12 cells injury: involvement of PI3 kinase signal pathway. Acta Pharmacol Sin (2009) 30(2):159–65. doi: 10.1038/aps.2008.25 PMC4002468 19151742

[212] Koo HJ Lim KH Jung HJ Park EH. Anti-inflammatory evaluation of gardenia extract, geniposide and genipin. J Ethnopharmacol (2006) 103(3):496–500. doi: 10.1016/j.jep.2005.08.011 16169698

[213] Hu X Zhang X Jin G Shi Z Sun W Chen F. Geniposide reduces development of streptozotocin-induced diabetic nephropathy *via* regulating nuclear factor-kappa b signaling pathways. Fundam Clin Pharmacol (2017) 31(1):54–63. doi: 10.1111/fcp.12231 27521287

[214] Anders HJ Muruve DA. The inflammasomes in kidney disease. J Am Soc Nephrol (2011) 22(6):1007–18. doi: 10.1681/ASN.2010080798 21566058

[215] Guo H Callaway JB Ting JP. Inflammasomes: mechanism of action, role in disease, and therapeutics. Nat Med (2015) 21(7):677–87. doi: 10.1038/nm.3893 PMC4519035 26121197

[216] Patel MN Carroll RG Galvan-Pena S Mills EL Olden R Triantafilou M. Inflammasome priming in sterile inflammatory disease. Trends Mol Med (2017) 23(2):165–80. doi: 10.1016/j.molmed.2016.12.007 28109721

[217] Callender DM Hsue G. Artesunate: investigational drug for the treatment of severe falciparum malaria in hawai’i. Hawaii Med J (2011) 70(4):77–9. PMC3072541 21785506

[218] Shakeri A Amini E Asili J Masullo M Piacente S Iranshahi M. Screening of several biological activities induced by different sesquiterpene lactones isolated from centaurea behen l. and rhaponticum repens (L.) hidalgo. Nat Prod Res (2018) 32(12):1436–40. doi: 10.1080/14786419.2017.1344661 28641489

[219] Verma S Kumar VL. Artesunate affords protection against aspirin-induced gastric injury by targeting oxidative stress and proinflammatory signaling. Pharmacol Rep (2018) 70(2):390–7. doi: 10.1016/j.pharep.2017.06.003 29397336

[220] Mehrotra E Vishwakarma J Tripathi AC Sonar PK Saraf SK. Schizonticidal antimalarial sesquiterpene lactones from magnolia champaca (L.) baill. ex Pierre: microwave-assisted extraction, HPTLC fingerprinting and computational studies. Nat Prod Res (2019) 33(4):568–72. doi: 10.1080/14786419.2017.1396595 29086620

[221] Zhang XL Jiang B Li ZB Hao S An LJ. Catalpol ameliorates cognition deficits and attenuates oxidative damage in the brain of senescent mice induced by d-galactose. Pharmacol Biochem Behav (2007) 88(1):64–72. doi: 10.1016/j.pbb.2007.07.004 17698178

[222] Liu Z Zhu P Zhang L Xiong B Tao J Guan W. Autophagy inhibition attenuates the induction of anti-inflammatory effect of catalpol in liver fibrosis. BioMed Pharmacother (2018) 103:1262–71. doi: 10.1016/j.biopha.2018.04.156 29864907

[223] Chen X Liao D Qin Z Li X. Synergistic interactions of catalpol and stachyose in STZ-HFD induced diabetic mice: synergism in regulation of blood glucose, lipids, and hepatic and renal function. Chin Herbal Medicines (2019) 11(1):70–7. doi: 10.1016/j.chmed.2018.05.006

[224] Dai Y Chen SR Chai L Zhao J Wang Y Wang Y. Overview of pharmacological activities of andrographis paniculata and its major compound andrographolide. Crit Rev Food Sci Nutr (2019) 59(sup1):S17–29. doi: 10.1080/10408398.2018.1501657 30040451

[225] Ji X Li C Ou Y Li N Yuan K Yang G. Andrographolide ameliorates diabetic nephropathy by attenuating hyperglycemia-mediated renal oxidative stress and inflammation *via* Akt/NF-kappaB pathway. Mol Cell Endocrinol (2016) 437:268–79. doi: 10.1016/j.mce.2016.06.029 27378149

[226] Gao J Zhang Y Liu X Wu X Huang L Gao W. Triptolide: pharmacological spectrum, biosynthesis, chemical synthesis and derivatives. Theranostics (2021) 11(15):7199–221. doi: 10.7150/thno.57745 PMC8210588 34158845

[227] Hou W Liu B Xu H. Triptolide: medicinal chemistry, chemical biology and clinical progress. Eur J Med Chem (2019) 176:378–92. doi: 10.1016/j.ejmech.2019.05.032 31121546

[228] Chen SR Dai Y Zhao J Lin L Wang Y Wang Y. A mechanistic overview of triptolide and celastrol, natural products from tripterygium wilfordii hook f. Front Pharmacol (2018) 9:104. doi: 10.3389/fphar.2018.00104 29491837 PMC5817256

[229] Ponikvar-Svet M Zeiger DN Liebman JF. Alkaloids and selected topics in their thermochemistry. Molecules (2021) 26(21):6715 doi: 10.3390/molecules26216715 34771124 PMC8587110

[230] Abookleesh FL Al-Anzi BS Ullah A. Potential antiviral action of alkaloids. Molecules (2022) 27(3):903. doi: 10.3390/molecules27030903 35164173 PMC8839337

[231] Tang LQ Wang FL Zhu LN Lv F Liu S Zhang ST. Berberine ameliorates renal injury by regulating G proteins-AC- cAMP signaling in diabetic rats with nephropathy. Mol Biol Rep (2013) 40(6):3913–23. doi: 10.1007/s11033-012-2468-0 23266672

[232] Wang YY Tang LQ Wei W. Berberine attenuates podocytes injury caused by exosomes derived from high glucose-induced mesangial cells through TGFbeta1-PI3K/AKT pathway. Eur J Pharmacol (2018) 824:185–92. doi: 10.1016/j.ejphar.2018.01.034 29378192

[233] Ni WJ Zhou H Ding HH Tang LQ. Berberine ameliorates renal impairment and inhibits podocyte dysfunction by targeting the phosphatidylinositol 3-kinase-protein kinase b pathway in diabetic rats. J Diabetes Investig (2020) 11(2):297–306. doi: 10.1111/jdi.13119 PMC7078081 31336024

[234] Srinivasan K. Black pepper and its pungent principle-piperine: a review of diverse physiological effects. Crit Rev Food Sci Nutr (2007) 47(8):735–48. doi: 10.1080/10408390601062054 17987447

[235] Haq IU Imran M Nadeem M Tufail T Gondal TA Mubarak MS. Piperine: a review of its biological effects. Phytother Res (2021) 35(2):680–700. doi: 10.1002/ptr.6855 32929825

[236] Manayi A Nabavi SM Setzer WN Jafari S. Piperine as a potential anti-cancer agent: a review on preclinical studies. Curr Med Chem (2018) 25(37):4918–28. doi: 10.2174/0929867324666170523120656 28545378

[237] Bailly C. Cepharanthine: an update of its mode of action, pharmacological properties and medical applications. Phytomedicine (2019) 62:152956. doi: 10.1016/j.phymed.2019.152956 31132753 PMC7126782

[238] Liang D Li Q Du L Dou G. Pharmacological effects and clinical prospects of cepharanthine. Molecules (2022) 27(24):8933. doi: 10.3390/molecules27248933 36558061 PMC9782661

[239] Wang X Zou S Lan YL Xing JS Lan XQ Zhang B. Solasonine inhibits glioma growth through anti-inflammatory pathways. Am J Transl Res (2017) 9(9):3977–89. PMC5622243 28979674

[240] Liang X Hu C Han M Liu C Sun X Yu K. Solasonine inhibits pancreatic cancer progression with involvement of ferroptosis induction. Front Oncol (2022) 12:834729. doi: 10.3389/fonc.2022.834729 35494004 PMC9039314

[241] Zhang H Yan L. Solasonine relieves sevoflurane-induced neurotoxicity *via* activating the AMP-activated protein kinase/FoxO3a pathway. Hum Exp Toxicol (2022) 41:9603271211069984. doi: 10.1177/09603271211069984 35350913

[242] Son JK Chang HW Jahng Y. Progress in studies on rutaecarpine. II.–synthesis and structure-biological activity relationships. Molecules (2015) 20(6):10800–21. doi: 10.3390/molecules200610800 PMC6272352 26111170

[243] Byun WS Bae ES Kim WK Lee SK. Antitumor activity of rutaecarpine in human colorectal cancer cells by suppression of wnt/beta-catenin signaling. J Nat Prod (2022) 85(5):1407–18. doi: 10.1021/acs.jnatprod.2c00224 35544614

[244] Jia S Hu C. Pharmacological effects of rutaecarpine as a cardiovascular protective agent. Molecules (2010) 15(3):1873–81. doi: 10.3390/molecules15031873 PMC6257227 20336017

[245] Shao BZ Xu HY Zhao YC Zheng XR Wang F Zhao GR. NLRP3 inflammasome in atherosclerosis: putting out the fire of inflammation. Inflammation (2023) 46(1):35–46. doi: 10.1007/s10753-022-01725-x 35953687

[246] Neelam Khatkar A Sharma KK. Phenylpropanoids and its derivatives: biological activities and its role in food, pharmaceutical and cosmetic industries. Crit Rev Food Sci Nutr (2020) 60(16):2655–75. doi: 10.1080/10408398.2019.1653822 31456411

[247] Kolaj I Imindu Liyanage S Weaver DF. Phenylpropanoids and alzheimer’s disease: a potential therapeutic platform. Neurochem Int (2018) 120:99–111. doi: 10.1016/j.neuint.2018.08.001 30098379

[248] Korkina LG. Phenylpropanoids as naturally occurring antioxidants: from plant defense to human health. Cell Mol Biol (Noisy-le-grand) (2007) 53(1):15–25. 17519109

[249] Lee HJ Cho IH Lee KE Kim YS. The compositions of volatiles and aroma-active compounds in dried omija fruits (Schisandra chinensis baillon) according to the cultivation areas. J Agric Food Chem (2011) 59(15):8338–46. doi: 10.1021/jf200762h 21682319

[250] Wei M Liu Z Liu Y Li S Hu M Yue K. Urinary and plasmatic metabolomics strategy to explore the holistic mechanism of lignans in s. chinensis in treating alzheimer’s disease using UPLC-Q-TOF-MS. Food Funct (2019) 10(9):5656–68. doi: 10.1039/c9fo00677j 31433414

[251] Pu H Qian Q Wang F Gong M Ge X. Schizandrin a induces the apoptosis and suppresses the proliferation, invasion and migration of gastric cancer cells by activating endoplasmic reticulum stress. Mol Med Rep (2021) 24(5):787. doi: 10.3892/mmr.2021.12427 34498719 PMC8441983

[252] Zong W Gouda M Cai E Wang R Xu W Wu Y. The antioxidant phytochemical schisandrin a promotes neural cell proliferation and differentiation after ischemic brain injury. Molecules (2021) 26(24):7466. doi: 10.3390/molecules26247466 34946548 PMC8706049

[253] Li X Wu J Xu F Chu C Li X Shi X. Use of ferulic acid in the management of diabetes mellitus and its complications. Molecules (2022) 27(18):6010. doi: 10.3390/molecules27186010 36144745 PMC9503003

[254] Neto-Neves EM da Silva Maia Bezerra Filho C Dejani NN de Sousa DP. Ferulic acid and cardiovascular health: therapeutic and preventive potential. Mini Rev Med Chem (2021) 21(13):1625–37. doi: 10.2174/1389557521666210105122841 33402085

[255] Zhang D Jing B Chen Z Li X Shi H Zheng Y. Ferulic acid alleviates sciatica by inhibiting peripheral sensitization through the RhoA/p38MAPK signalling pathway. Phytomedicine (2022) 106:154420. doi: 10.1016/j.phymed.2022.154420 36115115

[256] Thapliyal S Singh T Handu S Bisht M Kumari P Arya P. A review on potential footprints of ferulic acid for treatment of neurological disorders. Neurochem Res (2021) 46(5):1043–57. doi: 10.1007/s11064-021-03257-6 33547615

[257] Chowdhury S Ghosh S Das AK Sil PC. Ferulic acid protects hyperglycemia-induced kidney damage by regulating oxidative insult, inflammation and autophagy. Front Pharmacol (2019) 10:27. doi: 10.3389/fphar.2019.00027 30804780 PMC6371841

[258] Qi MY Wang XT Xu HL Yang ZL Cheng Y Zhou B. Protective effect of ferulic acid on STZ-induced diabetic nephropathy in rats. Food Funct (2020) 11(4):3706–18. doi: 10.1039/c9fo02398d 32307498

[259] Choi R Kim BH Naowaboot J Lee MY Hyun MR Cho EJ. Effects of ferulic acid on diabetic nephropathy in a rat model of type 2 diabetes. Exp Mol Med (2011) 43(12):676–83. doi: 10.3858/emm.2011.43.12.078 PMC3256295 21975281

[260] Zhou D He L. Sauchinone inhibits hypoxia-induced invasion and epithelial-mesenchymal transition in osteosarcoma cells *via* inactivation of the sonic hedgehog pathway. J Recept Signal Transduct Res (2022) 42(2):173–9. doi: 10.1080/10799893.2021.1881556 33563062

[261] Fisher L. Retraction: sauchinone inhibits high glucose-induced oxidative stress and apoptosis in retinal pigment epithelial cells. RSC Adv (2021) 11(9):5243. doi: 10.1039/d1ra90044g 35427013 PMC8694693

[262] Chae HS You BH Kim DY Lee H Ko HW Ko HJ. Sauchinone controls hepatic cholesterol homeostasis by the negative regulation of PCSK9 transcriptional network. Sci Rep (2018) 8(1):6737. doi: 10.1038/s41598-018-24935-6 29712938 PMC5928089

[263] Han HJ Li M Son JK Seo CS Song SW Kwak SH. Sauchinone, a lignan from saururus chinensis, attenuates neutrophil pro-inflammatory activity and acute lung injury. Int Immunopharmacol (2013) 17(2):471–7. doi: 10.1016/j.intimp.2013.07.011 23928505

[264] Amerizadeh F Rezaei N Rahmani F Hassanian SM Moradi-Marjaneh R Fiuji H. Crocin synergistically enhances the antiproliferative activity of 5-flurouracil through Wnt/PI3K pathway in a mouse model of colitis-associated colorectal cancer. J Cell Biochem (2018) 119(12):10250–61. doi: 10.1002/jcb.27367 30129057

[265] Godugu C Pasari LP Khurana A Anchi P Saifi MA Bansod SP. Crocin, an active constituent of crocus sativus ameliorates cerulein induced pancreatic inflammation and oxidative stress. Phytother Res (2020) 34(4):825–35. doi: 10.1002/ptr.6564 31769107

[266] Shafahi M Vaezi G Shajiee H Sharafi S Khaksari M. Crocin inhibits apoptosis and astrogliosis of hippocampus neurons against methamphetamine neurotoxicity *via* antioxidant and anti-inflammatory mechanisms. Neurochem Res (2018) 43(12):2252–9. doi: 10.1007/s11064-018-2644-2 30259275

[267] Sung YY Kim HK. Crocin ameliorates atopic dermatitis symptoms by down regulation of Th2 response *via* blocking of NF-kappaB/STAT6 signaling pathways in mice. Nutrients (2018) 10(11):1625. doi: 10.3390/nu10111625 30400140 PMC6266819

[268] Zhou Y Xu Q Shang J Lu L Chen G. Crocin inhibits the migration, invasion, and epithelial-mesenchymal transition of gastric cancer cells *via* miR-320/KLF5/HIF-1alpha signaling. J Cell Physiol (2019) 234(10):17876–85. doi: 10.1002/jcp.28418 30851060

[269] Samarghandian S Azimi-Nezhad M Farkhondeh T. Crocin attenuate tumor necrosis factor-alpha (TNF-alpha) and interleukin-6 (IL-6) in streptozotocin-induced diabetic rat aorta. Cytokine (2016) 88:20–8. doi: 10.1016/j.cyto.2016.08.002 27529541

[270] Yamada Y Nishii K Kuwata K Nakamichi M Nakanishi K Sugimoto A. Effects of pyrroloquinoline quinone and imidazole pyrroloquinoline on biological activities and neural functions. Heliyon (2020) 6(1):e03240. doi: 10.1016/j.heliyon.2020.e03240 32021931 PMC6994848

[271] Liu Z Sun C Tao R Xu X Xu L Cheng H. Pyrroloquinoline quinone decelerates rheumatoid arthritis progression by inhibiting inflammatory responses and joint destruction *via* modulating NF-kappaB and MAPK pathways. Inflammation (2016) 39(1):248–56. doi: 10.1007/s10753-015-0245-7 26319019

[272] Zhang Q Zhou J Shen M Xu H Yu S Cheng Q. Pyrroloquinoline quinone inhibits rotenone-induced microglia inflammation by enhancing autophagy. Molecules (2020) 25(19):4359. doi: 10.3390/molecules25194359 32977419 PMC7582530

[273] Jonscher KR Chowanadisai W Rucker RB. Pyrroloquinoline-quinone is more than an antioxidant: a vitamin-like accessory factor important in health and disease prevention. Biomolecules (2021) 11(10):1441. doi: 10.3390/biom11101441 34680074 PMC8533503

[274] Van den Worm E Beukelman CJ Van den Berg AJ Kroes BH Labadie RP Van Dijk H. Effects of methoxylation of apocynin and analogs on the inhibition of reactive oxygen species production by stimulated human neutrophils. Eur J Pharmacol (2001) 433(2-3):225–30. doi: 10.1016/s0014-2999(01)01516-3 11755156

[275] Touyz RM. Apocynin, NADPH oxidase, and vascular cells: a complex matter. Hypertension (2008) 51(2):172–4. doi: 10.1161/HYPERTENSIONAHA.107.103200 18086948

[276] Altenhofer S Radermacher KA Kleikers PW Wingler K Schmidt HH. Evolution of NADPH oxidase inhibitors: selectivity and mechanisms for target engagement. Antioxid Redox Signal (2015) 23(5):406–27. doi: 10.1089/ars.2013.5814 PMC4543484 24383718

[277] Heumuller S Wind S Barbosa-Sicard E Schmidt HH Busse R Schroder K. Apocynin is not an inhibitor of vascular NADPH oxidases but an antioxidant. Hypertension (2008) 51(2):211–7. doi: 10.1161/HYPERTENSIONAHA.107.100214 18086956

[278] Malla R Marni R Chakraborty A Kamal MA. Diallyl disulfide and diallyl trisulfide in garlic as novel therapeutic agents to overcome drug resistance in breast cancer. J Pharm Anal (2022) 12(2):221–31. doi: 10.1016/j.jpha.2021.11.004 PMC9091922 35582397

[279] Gong X Su X Liu H. Diallyl trisulfide, the antifungal component of garlic essential oil and the bioactivity of its nanoemulsions formed by spontaneous emulsification. Molecules (2021) 26(23):7186. doi: 10.3390/molecules26237186 34885768 PMC8658937

[280] Pang H Wang C Ye J Wang L Zhou X Ge X. Diallyl trisulfide plays an antifibrotic role by inhibiting the expression of bcl-2 in hepatic stellate cells. J Biochem Mol Toxicol (2022) 36(8):e23097. doi: 10.1002/jbt.23097 35532220 PMC9539501

